# Human MAIT cells undergo clonal selection and expansion during thymic maturation and aging

**DOI:** 10.1038/s12276-025-01509-x

**Published:** 2025-08-08

**Authors:** Myeong-seok Lee, Suyeong Park, Jung-Hwan Choi, Seon Yong Bae, Han Suk Ryu, Min-Sung Kim, Jae Gun Kwak, You Jeong Lee

**Affiliations:** 1 Research Institute of Pharmaceutical Sciences, College of Pharmacy, Seoul National University, Seoul, Republic of Korea; 2 Department of Thoracic and Cardiovascular Surgery, Seoul National University Hospital, College of Medicine, Seoul National University, Seoul, Republic of Korea; 3 Department of Pathology, Seoul National University Hospital, College of Medicine, Seoul National University, Seoul, Republic of Korea; 4 Department of Life Sciences, Pohang University of Science and Technology, Pohang, Republic of Korea

**Keywords:** T-cell receptor, VDJ recombination

## Abstract

Mucosal-associated invariant T (MAIT) cells harbor conserved T cell receptors (TCRs) recognizing riboflavin metabolites, yet exhibit substantial diversity similar to conventional memory T cells. However, the mechanisms shaping this diversity related to their thymic ontogeny remain unclear. Here we analyze 37 samples of human thymic MAIT cells across ages and compare them with other unconventional T cells, such as iNKT and γδ T cells. We find that CD27 and CD161 serve as common markers distinguishing the maturation stages of unconventional T cells such as MAIT, iNKT and Vγ9^+^Vδ2^+^ γδ T cells. Notably, CD161^+^ mature MAIT cells clonally expand proportionally to aging with the upregulation of genes associated with tissue residency. MAIT cell diversity is initially determined by diverse CDR3β sequences, which become reduced upon maturation. Furthermore, 25% of MAIT cells express polyclonal dual TCRα transcripts, suggesting they arise from double-positive thymocytes with random TCRα rearrangement. Collectively, these findings show that thymic MAIT cells undergo dynamic regulation of repertoire selection, similar to conventional T cells.

## Thymic MAIT cells reveal dynamic TCR diversity patterns

This study explores how certain immune cells, called MAIT cells, develop in the human thymus, an organ where immune cells mature. Researchers wanted to understand how these cells change as people age and how they compare to other similar immune cells. Researchers used advanced techniques such as single-cell RNA sequencing to study the genes and receptors of these cells. They found that MAIT cells with certain receptor sequences, called clonotypes, are selected to survive and mature in the thymus. The study also showed that MAIT cells undergo a process called clonal expansion, where certain clonotypes multiply more than others in adults. These features are not seen in other similar cells, which remain stable. Overall, these findings suggest that MAIT cells are uniquely shaped in the thymus and may play a unique role in the immune system as people age.

This summary was initially drafted using artificial intelligence, then revised and fact-checked by the author.

## Introduction

Human mucosal-associated invariant T (MAIT) cells have a canonical TRAV1-2 (T cell receptor (TCR) Vα7.2) chain paired with oligoclonal TRAJ33/20/12 and TRBV20/6 chains, recognizing bacteria-derived vitamin B2 metabolites in the context of MR1^1,2^. In mice, MAIT cells, similar to invariant natural killer T (iNKT) cells, and some γδ T cells develop in the thymus as memory cells expressing the transcription factor promyelocytic zinc finger (PLZF) protein^2,3^. In the human thymus, MAIT cells undergo sequential differentiation from CD27^−^CD161^−^ stage 1 to CD27^+^CD161^−^ stage 2 and finally to CD161^+^ stage 3 (ref. ^4^). Stage 3 mature MAIT cells co-express PLZF, TBET and RORγt and secrete IFNγ, TNFα and IL-17, indicating that serial differentiation accompanies the acquisition of innate phenotype^4^. Recent reports have shown that the clonal diversity of MAIT cells is comparable to that of conventional memory T cells^5^, and polyclonal MAIT cells are present in the periphery^6,7^. However, the mechanisms that shape receptor diversity during thymic development, where they acquire innate phenotypes, remain poorly understood. Furthermore, it is unclear whether MAIT cells undergo clonal selection or expansion in a manner similar to that of conventional memory T cells.

Recent studies have analyzed the transcriptional profiles of human MAIT cells during thymic development, revealing that MAIT and iNKT cells share conserved transcriptional programs across species^5,8–10^. iNKT cells have TRAV10 (TCR Vα24) paired with TRAJ18 and TRBV25-1 and are restricted by CD1d presenting lipid antigens^11–13^. Previously, we and others showed innate T cells in mice share common lineage differentiation programs generating type 1, type 2 and type 17 effector lineages^12,14,15^. In the thymus, human MAIT, iNKT and Vγ9^+^ Vδ2^+^ γδ T cells shared distinctive transcriptional programs distinguished from conventional T cells^10^. These studies, however, used human thymic tissues obtained from donors under 2 years old, and it is not well understood whether the developmental programs of MAIT and iNKT cells are stable for a lifetime or whether there are long-term tissue-resident populations formed similarly to mice natural killer T (NKT) cells^16^.

We address the above issues to fill the knowledge gap in the field. For this, we analyzed the thymic ontogeny of human counterparts of mouse innate T cells, focusing on repertoire selection of MAIT and iNKT cells using 37 postnatal thymi ranging from 2 weeks to 56 years of age. We sorted MAIT, NKT and γδ T cells from four samples representing different age groups and performed single-cell RNA sequencing (scRNA-seq) combined with TCR repertoire analysis. This approach enabled us to uncover their ontogeny, clonal selection and maturation pathways across age groups. We found that CD27 and CD161 are effective markers for delineating the developmental stages of MAIT and iNKT cells. While mature and immature MAIT and iNKT cells broadly shared transcriptional similarities, they exhibited distinct maturation patterns. By constrat, most of the classical γδ T cells were markedly different in their transcriptional profiles. Intriguingly, only CD8^+^ MAIT cells efficiently matured into stage 3 cells, while iNKT cells predominantly remained immature at stage 2.

In the TCR repertoire analysis, thymic MAIT and iNKT cells utilized canonical CDR3α sequences paired with diverse CDR3β sequences, generating diverse clonotypes from an immature stage. Notably, mature MAIT cells in the adult human thymus underwent clonal expansion and upregulated genes associated with tissue residency. During the MAIT cell maturation, we also observed a reduction in TCR Vβ diversity, indicating that the selection of semi-invariant TCRs is required for their final maturation or long-term residence. Moreover, approximately 25% of human MAIT and iNKT cells possessed an additional polyclonal TCRα transcript, similar to conventional T cells, suggesting they arise from random TCR gene rearrangement in double-positive (DP) thymocytes. However, polyclonal TRAV1-2-negative noncanonical MAIT cells were not detected in the thymus when we examined 27 clonotypes. Collectively, our findings show that the diverse repertoire of human MAIT cells is established in the thymus by pairing public TCRα chain with private TCRβ chains, and their maturation accompanies clonal selection and expansion similar to conventional memory T cells.

## Materials and methods

### Study design

In this study, we investigated the maturation pathways of innate T cells across different ages using a cohort of 37 postnatal thymi. MAIT, iNKT and γδ T cells were obtained via magnetic-based and flow-based cell sorting from thymocytes of four donors spanning various age groups. Their gene expression profiles and clonotypes were analyzed using scRNA-seq along with TCR repertoire analysis. We cloned 27 random polyclonal TCRs into a lentiviral vector to test the presence of polyclonal MAIT cells.

### Human samples

Human thymi and blood samples were obtained from patients who underwent cardiac surgery. Thymectomy was performed only in cases where thymic tissue interrupted operative fields, and its removal was kept to a minimum to improve exposure during corrective cardiovascular surgery. As previously described^17,18^, human thymocytes and peripheral blood mononuclear cells (PBMCs) were isolated by density gradient centrifugation using Ficoll-Paque (GE Healthcare Life Science). This study was reviewed and approved by the Seoul National University Hospital (Seoul, Republic of Korea; H-2206-221-1337) and conducted according to the principles of the Declaration of Helsinki. Informed consent was obtained from all study participants.

### Flow cytometry

Biotinylated PBS57-loaded CD1d monomers and 5-OP-RU-loaded MR1 monomers were obtained from the tetramer facility of the US National Institutes of Health. Tetramer synthesis was described previously^19^. Single-cell suspensions were surface stained, fixed and permeabilized for intracellular staining with an eBioscience Foxp3 staining buffer set as described^20,21^. Cells were analyzed on LSR Fortessa X-20 (BD Biosciences), and data were processed with FlowJo software (TreeStar).

### Cell sorting for scRNA-seq

As previously described^22^, single-cell suspensions of human thymocytes were stained with BV421-conjugated anti-human TCRγδ or PE-conjugated CD1d, and APC-conjugated MR1 tetramers after blocking with anti-CD36 antibody^23^, then enriched with anti-PE and anti-APC microbeads (Miltenyi) according to the manufacturer’s instructions. After sorting, iNKT, MAIT and γδ T cells were mixed and analyzed for single-cell sequencing.

### TCR transfection assay

Based on the previous protocol^24^, synthetic TCRα and TCRβ sequences from MR1 tetramer-bound sorted single T cells were cloned into a pLV-EF1α-Puro vector (Thermo Fisher Scientific). Human embryonic kidney (HEK293T) cells were cultured overnight on a 24-well plate containing 300 μl of Dulbecco’s modified Eagle medium supplemented with 10% fetal bovine serum and penicillin–streptomycin at 37 °C, and they were subsequently transfected with the pLV-TCR plasmid using FuGENE HD transfection reagent (Promega). Transfected HEK293T cells were analyzed for tetramer binding by flow cytometry 48 h after transfection.

### 10x Genomics library generation and sequencing

Sequencing libraries were generated using 10x Genomics Chromium Single Cell 5′ Reagent Kits (v2 Chemistry Dual Index, following the manufacturer’s instructions). Cells were loaded onto the Chromium Controller (10x Genomics) at a concentration of approximately 0.5 × 10^6^ cells/ml, with 8000–10,000 cells targeted for sequencing. Library quality and concentration were assessed using a TapeStation (Agilent) and Countess IIFL Automated Cell Counter (Thermo Fisher Scientific), respectively. Library generation and sequencing on an Illumina NovaSeq6000 were performed at Geninus. Sequencing depths were 29,860–32,634 reads per cell for scRNA-seq.

### 10x Genomics raw data processing

FASTQ files were produced by converting BCL files with Illumina bcl2fastq. The gene expression and TCR data from these FASTQ files were analyzed using Cell Ranger (v.6.1.2) for both gene expression and TCR data. Specifically, for TCR analysis, the ‘filtered_contig_annotations.csv’ file was refined to include only high-confidence, complete and productive contigs associated with either TCRα or TCRβ chains.

### Quality control

Quality control was performed separately for cells from each channel of the Chromium Controller. Filtered feature–barcode matrices from Cell Ranger count were imported into R using Seurat^25^ (v.4.3.0.1). Cells with low gene counts and/or a high percentage of mitochondrial reads were removed. Furthermore, cells identified as potential doublets were excluded from the dataset for subsequent analysis. The probability of being a doublet was calculated using the pDoublet values, which were computed with the Scrubelt algorithm^26^ (v.0.1, python3). The cutoff values for pDoublet, nFeature_RNA and percent.mito are described in Supplementary Fig. 3. Cells expressing nontarget cell markers were excluded from further analysis. γδ T cells were validated with TRDC (T cell receptor delta constant) expression greater than 0.5, and the absence of a TCRαβ chain sequence NKT and MAITs were distinguished on the basis of TCRαβ chain information. Cells with no TRAC (T cell receptor alpha constant) and/or TRBC (T cell receptor beta constant) were excluded.

### Normalization, integration, dimensionality reduction and clustering

For the combined analysis of four different human thymus samples, data from each donor were independently normalized using the NormalizeData function in Seurat. Highly variable genes were defined as the top 2000 genes with the highest standardized variance, utilizing the FindVariableFeatures function in Seurat. Furthermore, TCR and Ig genes were removed from highly variable genes and subsequently normalized using the ScaleData function in Seurat to prevent any impact on subsequent clustering analyses pertaining to TCR or Ig gene utilization. Principal component analysis was used for dimensionality reduction, and the donor-specific batch correction was executed using Harmony^27^ (v0.1.1) considering 30 input principal components. ElbowPlots were consulted to determine the optimal number of principal components for UMAP construction and clustering. Cell clusters were delineated using Seurat’s graph-based clustering method.

### Cell type annotation

Only the subset of cells containing TCR sequences in scRNA-seq data was analyzed using the scRepertoire^28^ (v.1.7.0). Within this subset, cells were classified as either MAIT or NKT cells based on the usage of canonical Va chain genes, specifically TRAV1-2 or TRAV10, as determined by the TCR sequence data. Cells that did not express these genes were categorized as polyclonal cells. Three distinct maturation stages were determined on the basis of IL18R1, KLRB1 and CD27 expression using Seurat clustering. The distribution of cell types corresponding to each stage was visualized using the dittoBarPlot function of dittoSeq^29^ (v.1.6.0).

### Heterogeneity analysis (GEX)

The analysis was focused exclusively on the subset of cells containing TCR sequences in the scRNA-seq data, and the analysis was performed using the CombineTCR function from scRepertoire. Within the functionality of the CombineTCR function, we removed cells with missing TCR information (NA) and filtered multiplets, selecting the row with the highest read counts for multi TCRα or TCRβ as the final value.

### Differentially expressed gene (DEG) analysis

To assess the overall expression patterns of the cells, we used the plot_density function of Nebulosa^30^ (v.1.4.0). DEGs that exhibit differential expression between the comparison groups were identified using Seurat’s FindMarkers function. Subsequently, these DEGs were visualized utilizing the EnhancedVolcano function from EnhancedVolcano (v.1.13.2). The cutoff criteria for this analysis included a *P*-value threshold of 10^−5^ and a fold change threshold of 1. For the heat map analysis, DEGs were grouped on the basis of their shared functionality. Enriched gene lists were obtained from each group under comparison. Subsequently, heat map analysis was conducted using the Seurat’s DoHeatmap function. The signature score analysis was conducted using the Addmodulescore_UCell function of the UCell^31^ (v.1.3.1)

### Pathway analysis

Based on the list of DEGs, pathway analyses were conducted using Hallmark pathways from msigdbr (v.7.5.1) and Gene Ontology (GO): Biological Process 2023 from EnrichR^32^ (v.3.2). In the Hallmark pathway analysis, an initial Wilcoxon rank-sum test was performed on all genes using the wilcoxauc function of presto^33^ (v.1.0.0). Genes were ranked in descending order of their AUC score concerning the target group. Subsequently, the fgsea function from fgsea^34^ (v.1.20.0) was used to compute the normalized enrichment score and adjusted *P* value for the genes comprising each pathway based on their ranks. Hallmark pathways with adjusted *P* values less than 0.05 were presented. For the GO: Biological Process 2023 analysis, enriched pathways between groups were compared on the basis of the sorted rank values. This was achieved using EnrichR’s DEenrichRPlot function, which utilized the Wilcoxon rank-sum test. A log fold change threshold of 0.4 and a *P*-value threshold of 0.05 were used to identify the top ten GO: BP 2023 pathways based on log10pval. For biologically relevant pathways, the plotEnrichment function of fgsea was used to create gene set enrichment analysis plots for visualization.

### Pseudotime analysis

To utilize Monocle3^35,36^ (v.1.3.1), the initial step involved converting the Seurat object into a cell dataset using the as_cell_data_set function from SeuratWrappers (v.0.3.1). Then, the LearnGraph function was used to ascertain the graph structure within the reduced dimension space. The node representing the immature stage was subsequently set as the root using the order_cells function. Lastly, pseudotime values were depicted on UMAP embeddings using the plot_cells function.

### TCR repertoire analysis

All TCR analyses were conducted on the subset of cells with available gene expression information.

### TCR chain gene usage

To analyze TCR gene usage, the scRepertoire’s Vizgenes function was used to create heat maps that visualize TCR gene usage across patients. The data were scaled on the basis of the highest frequency among individual patients and cells. In addition, the ChorDiagram function from Circlize^37^ (v.0.4.15) was used to visualize the composition of TRAJ and TRBV by cell type and their interconnections, providing insights into the relationships between different cell types in the TCR repertoire.

### CDR3 amino acid sequence

The length distribution of CDR3 amino acids in TCRα and TCRβ was assessed using scRepertoire’s lengthContig function. Subsequently, sequence logos were visualized for amino acid sequences of a specific length with a single peak for each patient using Weblogo^38^ (v.2.8.2).

### Clonality analysis

TCR gene usage and matching CDR3 nucleotide sequences were designated as the same clone. Clones were categorized into groups based on their frequency of occurrence: single, small, medium, large and hyperexpanded, representing clone repeat counts of 1, 2–5, 6–20, 21–100 and 101–500, respectively. The Shannon index was computed using scRepertoire’s clonalDiversity function.

### Plots

Plots were generated with the ggplot224 (v.3.4.2). Fluorescence-activated cell sorting (FACS) plots were produced using FlowJo (v.10.9.0 by BD Biosciences). The other plots were constructed using GraphPad Prism (v.10.0.03 by GraphPad Software, LLC).

### Statistical analyses

Statistical analysis was performed using Microsoft Excel and GraphPad Prism version 10.1.1. Values reported in the figures are expressed as the standard error of the mean (s.e.m.), unless otherwise indicated. For normally distributed datasets, we used the paired-sample *t*-test between two comparison groups and one-way analysis of variance among three or more groups. *P* values >0.05 were considered not significant (ns), and *P* values <0.05 were considered significant. *P < 0.05, **P < 0.01, ***P < 0.001.

## Results

### Mature MAIT cells expand in the thymus with age

**Fig. 1:**
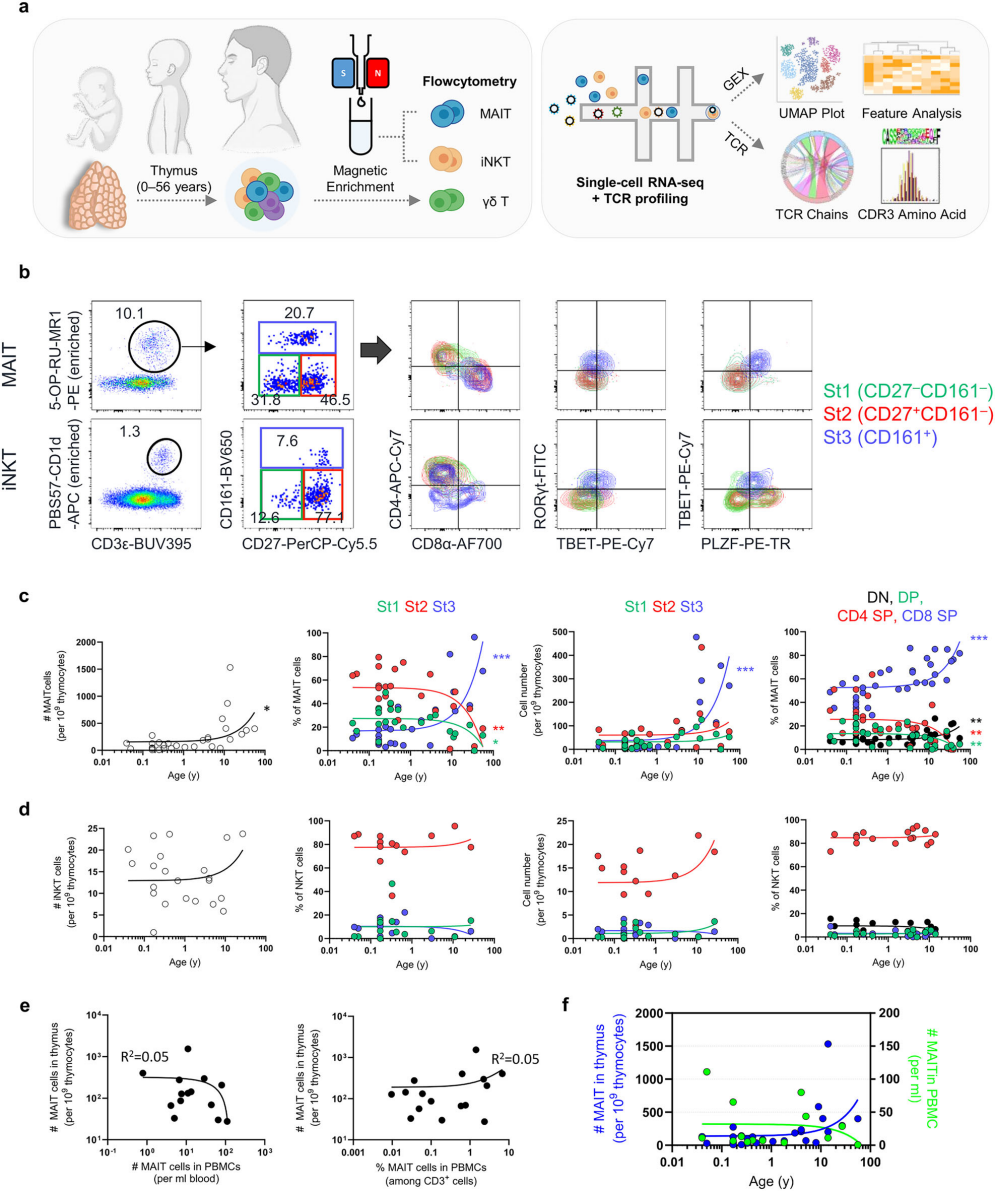
Mature MAIT cells expand in the thymus with age. **a** The schematic figure shows experimental procedures for isolating MAIT, iNKT and γδ T cells for flow cytometry (*n* = 37), and scRNA-seq combined with TCR sequencing from the thymus (*n* = 4). **b** Dot plots show representative flow cytometry analysis of human thymus, enriched with 5-OP-RU-loaded MR1 and PBS57-loaded CD1d tetramers. **c**, **d** The graphs depict the Pearson correlation analyses of the frequencies and numbers of MAIT (**c**) and iNKT (**d**) cells across the donors’ ages. **e** The graphs depict the correlations between the frequencies of MAIT cells in the thymus and their matched PBMC samples. **f** The graph shows the absolute numbers of MAIT cells in the thymus and PBMC in various age groups. The lines in the graphs represents the simple linear regression. Numbers indicate frequencies of cells in adjacent gates. St stage, SP single positive, DP double positive. *P < 0.05, **P < 0.01, ***P < 0.001.

To gain comprehensive insights into the thymic ontogeny of human MAIT cells, we analyzed 37 postnatal human thymi and simultaneously analyzed iNKT and γδ T cells for comparison (Fig. 1a). We enriched MAIT and iNKT cells using MR1 and CD1d tetramers or stained whole thymocytes with anti-TCRγδ antibodies (Supplementary Fig. 1a,b). Analogous to previous studies showing that CD27 and CD161 distinguish stages 1, 2 and 3 MAIT cells^4^, we found that iNKT cells follow a similar maturation pattern to MAIT cells in the thymus (Fig. 1b) and periphery (Supplementary Fig. 1c). Based on this, we designate stage 1 iNKT cells as CD27^−^CD161^−^, stage 2 cells as CD27^+^CD161^−^ and stage 3 cells as CD161^+^ (Fig. 1b, middle).

Unlike MAIT cells, iNKT cells upregulated PLZF in stages 1 and 2, and subsequently co-expressed PLZF, TBET, and RORγt, similar to MAIT cells in stage 3 (Fig. 1b). CD4⁺ iNKT cells have previously been characterized as immature populations in both the thymus and periphery^39^. The CD4 and CD161 combination was used to distinguish stage 1 (CD4^+^CD161^−^), stage 2 (CD4^−^CD161^−^) and stage 3 (CD4^−^CD161^+^) Vγ9^+^Vδ2^+^ γδ T cells^40^. Therefore, it is possible that iNKT cells follow a developmental scheme similar to that of Vγ9^+^Vδ2^+^ γδ T cells. To check this, we compared CD27–CD161 and CD4–CD161 classification frameworks in iNKT and MAIT cells (Supplementary Fig. 2a). Under the CD4–CD161 scheme, however, stage 1 iNKT cells upregulated PLZF, followed by downregulation at stage 2 and subsequent reexpression at stage 3, which is unlikely to reflect a physiologically relevant maturation trajectory. Given the coinciding expression of CD4 and CD27 in iNKT cells (Supplementary Fig. 2a), CD4⁺CD161^−^ iNKT cells are probably indicative of stage 2 cells.

Interestingly, the proportion of MAIT cells, but not iNKT cells, in the human thymus gradually increased with age, mainly due to the expansion of CD8 single-positive (SP) stage 3 cells (Fig. 1c). However, the frequency of stage 1 cells among total thymocytes remained stable across ages, suggesting that immature MAIT cells are continuously generated (Fig. 1c). By contrast, iNKT cells were arrested at stage 2, and the proportions of cells in each stage remained stable up to the mid-50s (Fig. 1d). The expansion of mature MAIT cells in the thymus was unrelated to the periphery, suggesting that thymic expansion is more likely to be driven by novo changes rather than systemic effects (Fig. 1e, f).

γδ T cells are distinguished by their expression of TCR chains, and fetal-derived Vγ9^+^Vδ2^+^ γδ T cells represent the CD161^+^ PLZF^+^ TBET^+^ innate subset, which is infrequently detected in postnatal thymus (Supplementary Fig. 3a,b). These cells were stable in the postnatal thymi, similar to iNKT cells (Supplementary Fig. 3c). Most of the non-Vγ9^+^Vδ2^+^ γδ T cells were CD161^−^ and did not express PLZF in the thymus (Supplementary Fig. 3), indicating that these are not innate T cells. Later, we referred to these cells as classic γδ T cells. Collectively, these features indicate that MAIT cell maturation is partly shared with, but distinct from, iNKT and γδ T cell maturation processes. Moreover, mature MAIT cells expand in the thymus in an age-dependent manner.

### Age-dependent kinetics of MAIT cell differentiation at the single-cell level

**Fig. 2:**
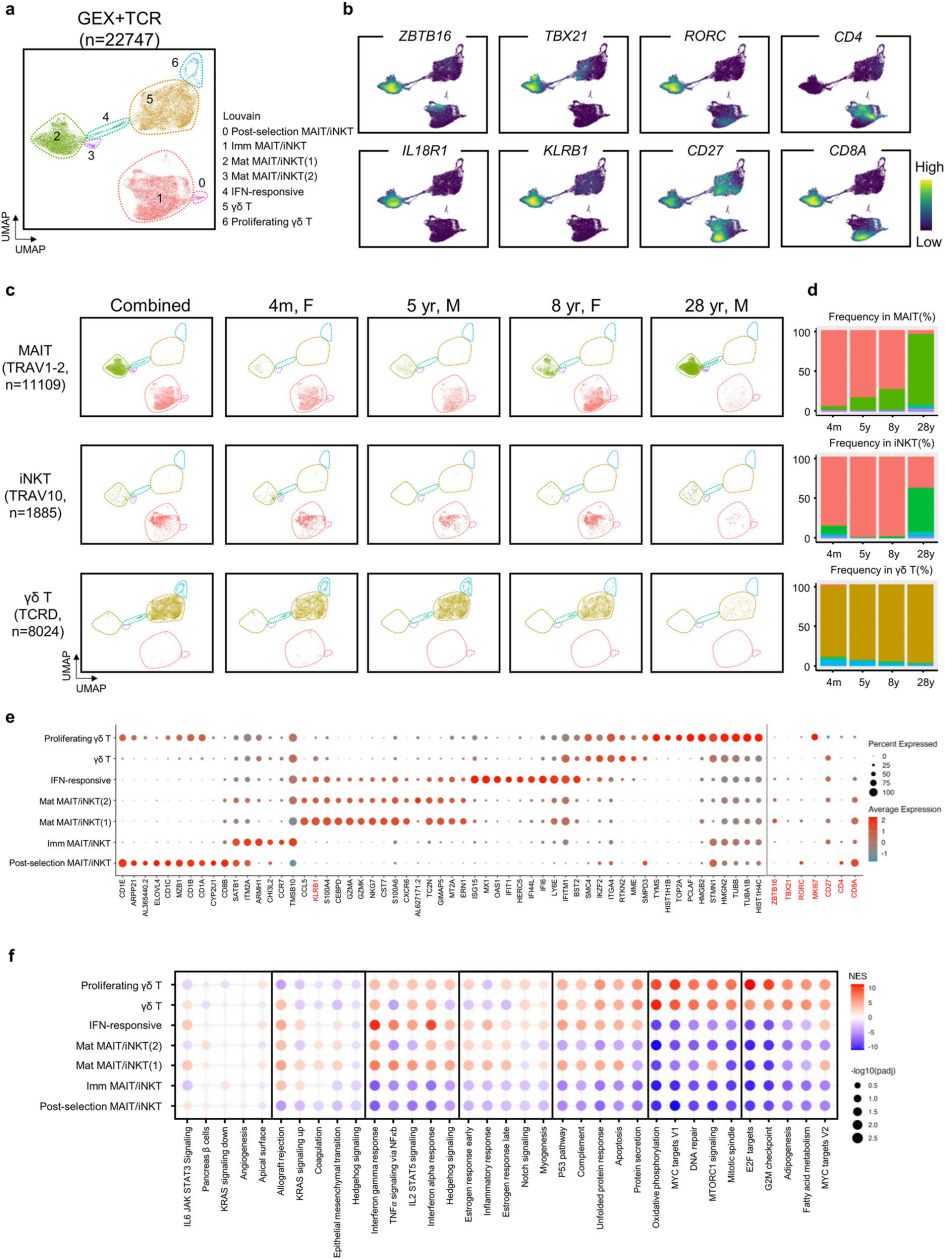
Age-dependent kinetics of MAIT cell differentiation at the single-cell level. **a** The UMAP plot shows combined NKT, MAIT and γδ T cells with visualization based on the Louvain clusters. **b** The density plots illustrate the expression of indicated markers. **c** The UMAP plot in **a** was separated by donors and cell types. **d** Frequency plots depict the distribution of stages across individual donors and indicated cell types. **e**, **f** The dot plots show each Louvain cluster’s top ten DEGs and manually selected markers (red) (**e**) and top five enriched pathways (**f**).

To gain further insights into the age-dependent expansion of human MAIT cells, we simultaneously sorted MAIT, iNKT and γδ T cells and performed scRNA-seq combined with TCR repertoire analysis, as outlined in Fig. 1a and Supplementary Fig. 1a. To encompass various age groups, we collected cells from four donors—a 5-month-old female, a 5-year-old male, an 8-year-old female and a 28-year-old male—yielding a total of 29,117 cells (21,153 αβ T cells and 8024 γδ T cells). We obtained αβ TCR information from 14,723 cells (a 69.6% detection rate) (Fig. 2a), following quality control (Supplementary Fig. 4a–e). Notably, 2383 cells (16.2%) displayed dual TCRα transcripts. We annotated cell types on the basis of the transcript with higher expression for these cells and identified MAIT (*n* = 11,109), iNKT (*n* = 1885) and polyclonal αβ T cells (*n* = 1729) (Supplementary Fig. 4f). For uniform manifold approximation and projection (UMAP) analysis, TCR genes were excluded from the DEG list to avoid TCR-dependent clustering. Seven distinct clusters (C0 to C6) were obtained (Fig. 2a). Supplementary Fig. 5 shows the distribution of cells in each donor, and sequence information is provided in Supplementary Table 1.

Previous studies have shown that MAIT and iNKT cells share similar transcriptional profiles in the thymus of individuals younger than 2 years old^9,10^. Our analysis confirmed that these cell types can be distinguished by their TCRs but not by their clustering in aged samples across ages, despite the expansion of mature cells observed in adult samples (Fig. 2a–d). In the UMAP, clusters C1 and C2 represented immature and mature stages of both MAIT and iNKT cells, distinguished by the simultaneous expression of PLZF (encoded by *ZBTB16*), TBET (encoded by *TBX21*), RORγt (encoded by *RORC*), IL18R1 (encoded by *IL18R1*) and CD161 (encoded by *KLRB1*) (Fig. 2b). Because we used postnatal thymic, we obtained only about 50 Vγ9^+^Vδ2^+^ γδ cells (data not shown), and the most of classical γδ T cells were not positioned together with MAIT or iNKT cells in UMAP. Consistent with the flow cytometric analysis (Fig. 1b–d), the proportions of mature CD8 SP MAIT cells expanded with aging (Fig. 2d). We generated a dot plot to display key markers of each cluster (Fig. 2e), along with a dot plot depicting the top five differentially upregulated pathways in each Louvain cluster (Fig. 2f). These dot plots further support that each cluster represents unique cell types, and classical γδ T cells are distinct from MAIT and iNKT cells. Cluster C0 represents postselection DP MAIT or iNKT cells, and its signature genes overlap with those of C6, which highly expresses Ki-67 (Fig. 2e). Therefore, we designated C6 as stage 0 proliferating immature γδ T cells. Overall, these findings indicate that, while the developmental pathways of MAIT and iNKT cells are similar, only mature MAIT cells expand with age, maintaining their innate phenotype.

### MAIT cells are distinguished from iNKT cells for their detailed maturation processes

**Fig. 3:**
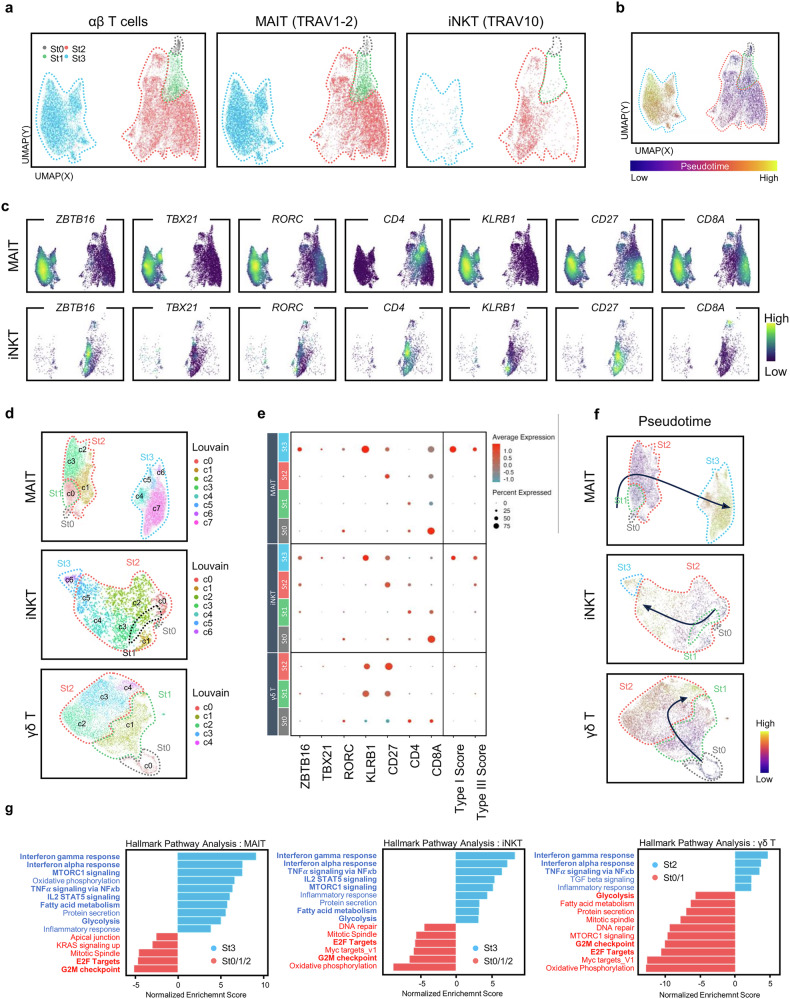
MAIT cells are distinguished from iNKT cells for their detailed maturation processes. **a**–**c** Combined analysis of MAIT and iNKT cells. UMAP plots visualize the total, MAIT and iNKT cells (**a**). The density plots illustrate pseudotime progression (**b**) and the expression levels of indicated markers (**c**). **d** UMAP plots display unique cell clusters in each cell type. Stages were annotated according to CD27 and CD161 expression patterns. **e** Dot plots show the differential expression of the indicated markers and type-immunity-related markers for each Louvain cluster of each cell type. **f** Pseudotime analysis shows the maturation pathways of each cell type. **g** Bar graphs display the enriched pathways for mature cells (blue) compared with immature cells (red), with an adjusted *P* value of less than 0.05. St, stage.

We separated MAIT and iNKT cells on the basis of TCR annotation and performed UMAP analysis (Fig. 3a–c). Consistent with flow cytometric analysis, CD27 and CD161 differentiated stage 1, 2 and 3 cells in the UMAP, and stage 0 postselection DP cells corresponded to C0 in Fig. 2 (Fig. 3a, c). Pseudotime analysis supported serial differentiation of MAIT and iNKT cells from stage 0 to stage 3 (Fig. 3b). In the density plots, we compared the expression patterns of various markers between MAIT and iNKT cells, revealing distinct developmental patterns between the two cell types (Fig. 3c). Specifically, iNKT cells co-expressed CD4 and CD27 in the immature cluster and immature iNKT cells prematurely upregulated PLZF before maturation, unlike MAIT cells. These features are consistent with FACS analyses in Fig. 1b and Supplementary Fig. 2 and support the idea that CD4 is not a marker of the most immature iNKT cells.

A heat map for MAIT and iNKT cells showed markers associated with cytokine, chemokine, cytotoxicity, chemokine and TCR signaling differentially expressed between each stage (Supplementary Fig. 6a). Mature MAIT and iNKT cells had higher expressions of genes associated with cytokine, chemokines and cytotoxic molecules, whereas immature cells upregulated genes associated with TCR signaling (Supplementary Fig. 6a). We further validated the expressions of HLA-ABC, GZMB, GZMK, CCR7 and TOX by flow cytometry (Supplementary Fig. 6b).

For more detailed analysis, we generated UMAPs for each cell type (Fig. 3d). In MAIT cells, we identified eight clusters, which could be grouped into two main categories: one composed of stage 0, 1 and 2 cells (C1, C2, C3 and C7), and the other composed of stage 3 cells (C0, C3, C4 and C6) expressing *PLZF*, *TBX21*, *RORC*, *IL18R1*, *KLRB1, CD8*, *PRF1* and *CD27* (Fig. 3d, e, top). This pattern was clearly illustrated in a heat map (Supplementary Fig. 7, left) and a volcano plot, which showed that stage 1 and 2 MAIT cells had only a few DEGs (Supplementary Fig. 8a, top). For a more robust analysis, we integrated two public datasets (GSE239558 and GSE189485), which contained respective 5994 and 1,1316 human MAIT cells in the thymus^8,9^ (Supplementary Fig. 9). The expression patterns of key markers in immature and mature cells, along with pseudotime analysis, confirmed consistent maturation trajectories across all three datasets (Supplementary Fig. 9).

In iNKT cells, we identified seven clusters, in which CD161^+^ mature cells (C6) were adjacent to clusters containing immature cells expressing *CD4* and *CD27* (Fig. 3d, e, middle), consistent with the flow cytometric analysis (Fig. 1b). Similar to MAIT cells, stage 1 and 2 iNKT cells had relatively few DEGs, while stage 3 cells were more distinct (Supplementary Fig. 8a, middle).

In classical γδ T cells, five clusters were identified (C0 to C4), corresponding to DP stage 0 (C0), CD27^+^ stage 1 cells (C1) and CD161^dim^ stage 2 cells (C2 to C4) (Fig. 3d, e, bottom). Pseudotime analysis supported the differentiation of C0 (DP, stage 0) cells into C1 (CD27^−^CD161^−^ stage 1) cells and C2 to C4 (CD27^+^ CD161^dim^ stage 2) cells (Fig. 3d–f, bottom). Volcano plots also revealed a substantial number of DEGs between stages 0, 1 and 2 (Supplementary Fig. 8a, bottom). However, CD161 expression on γδ T cells was lower than MAIT and iNKT cells (Fig. 2b), consistent with the CD161^dim^ pattern observed in flow cytometry of non-Vγ9^+^Vδ2^+^ γδ T cells (Supplementary Fig. 3a). These findings suggest that CD161 expression also marks the maturation of γδ T cells, albeit at lower levels than in MAIT or iNKT cells. As we used postnatal thymi, most of the γδ T cells were not Vγ9^+^Vδ2^+^, which are fetal-derived cells sharing a transcriptional nature with MAIT and iNKT cells^10^. Non-Vγ9^+^Vδ2^+^ γδ T cells were distinct from MAIT or iNKT cells in the UMAP and did not fully acquire markers such as PLZF, TBET and RORγt, suggesting they do not belong to the innate lineage (Fig. 2).

Pathway analyses revealed that MAIT and iNKT cells shared common maturation pathways (Fig. 3g and Supplementary Fig. 8b). In a Hallmark pathway analysis, mature MAIT and iNKT cells, compared with immature counterparts, upregulated pathways such as MTORC1 response, TNFα signaling, IL2-STAT5 signaling, fatty acid metabolism and glycolysis, while they downregulated E2F targets and G2M checkpoints (Fig. 3g). In GO pathway analysis, immature MAIT and iNKT cells were enriched in pathways related to antigen receptor-mediated signaling and T cell differentiation within the thymus, while mature cells upregulated pathways associated with cytokine-mediated signaling and cytotoxicity (Supplementary Fig. 8b). However, classical γδ T cells were not enriched with ‘cytokine-mediated signaling in mature cells’, and ‘T cell differentiation in thymus’ in immature cells, again suggesting they follow different maturation pathways (Supplementary Fig. 8b).

Collectively, these analyses indicate MAIT and iNKT cells share common maturation patterns, but are distinct in their detailed maturation processes.

### MAIT cells with canonical TCRs are selected upon maturation

**Fig. 4:**
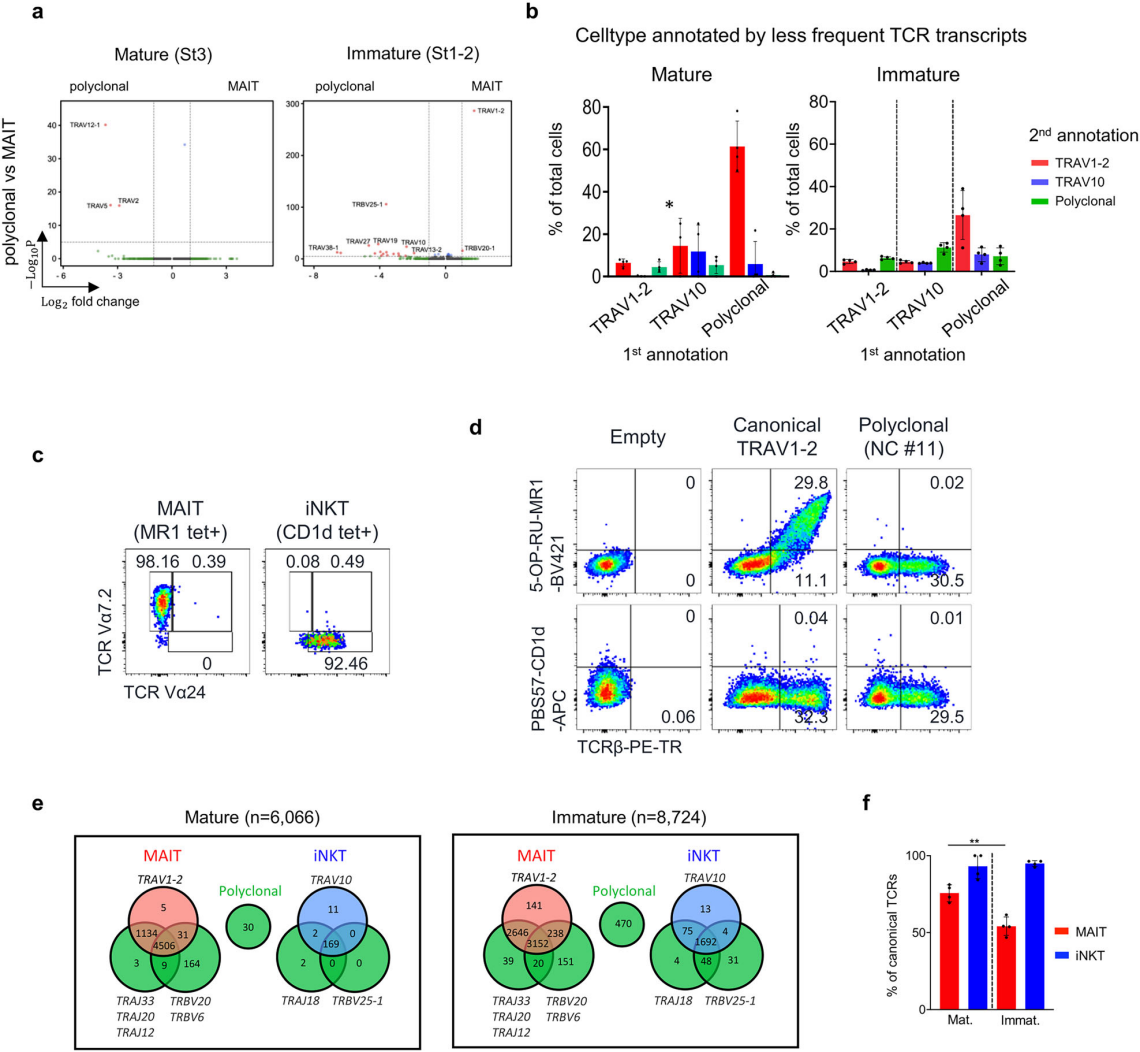
MAIT cells with canonical TCRs are selected upon maturation. **a** Volcano plots display the DEGs between MAIT and polyclonal cells in mature and immature stages. **b** Graphs show the frequency of cell type shifts when annotated with less frequent TCR transcripts in mature and immature cells. *One sample with four cells was excluded from the analysis. **c** Total thymocytes were enriched using 5-RO-RU-loaded MR1 or PBS57-loaded CD1d tetramer, followed by staining with anti-TCR Vα7.2 and Vα24 antibodies. **d** HEK293T cells were transfected with empty vectors, canonical MAIT TCRs (MAITc) or polyclonal TCRs (NC, noncanonical) and stained with MR1 and CD1d tetramers. Representative dot plots show a representative example of 27 NC samples. **e** Venn diagrams show the number of cells using the indicated combination of TCR chains. **f** The graph shows the frequency cells with all canonical combinations of TRAV, TRAJ and TRBV chains among total MAIT (TRAV1-2^+^) or iNKT (TRAV10^+^) cells. Numbers indicate the frequency of cells in each quadrant or adjacent gates. Error bars indicate s.e.m. **P < 0.01.

We obtained 1729 polyclonal αβ T cells from single-cell sequencing in both mature and immature cell clusters (Supplementary Fig. 5), displaying highly diverse usage of TCR genes (Supplementary Fig. 10). Because these cells had a minimal number of DEGs with canonical MAIT cells except TCR genes (Fig. 4a), we considered the dual TCRα transcripts, previously reported in 15% of human thymocytes^41^, and MR1 tetramer^low^ peripheral MAIT cells^42^. Supplementary Fig. 4g shows that an average of 50% of polyclonal T cells have second TCRα transcripts, and further analysis confirms that 65% of mature cells and 30% of immature cells have TRAV1-2 or TRAV10 as minor transcripts (Fig. 4b). Given that the detection rate for the TCRα gene in single-cell sequencing is about 70%, we estimate that 92% of mature cells and 42% of immature cells of polyclonal T cells have canonical MAIT or iNKT TCRs.

Notably, 10% of TRAV10^+^ iNKT cells expressed TRAV1-2 as minor transcripts (Fig. 4b). However, flow cytometry analysis showed that very few cells co-expressed TCR Vα7.2 (TRAV1-2) and Vα24 (TRAV10) (Fig. 4c), suggesting that only one of the dual TCR transcripts is expressed on the cell surface. Therefore, there remains the possibility that some polyclonal TCRs represent polyclonal MAIT or iNKT cells in the thymus, as a previous report identified TRAV36^+^ noncanonical MAIT cells in the periphery^7^. To convincingly test this possibility, we cloned 28 TCRs, 27 random polyclonal ones and 1 canonical MAIT one, into a lentiviral vector (Supplementary Table 2) and expressed them in HEK293T cells to assess their MR1 and CD1d tetramer binding (Fig. 4d and Supplementary Fig. 11). We confirm that none of the 27 TCRs showed binding to the tetramers, demonstrating that these TCRs do not recognize MR1 or CD1d. These results demonstrate that polyclonal TCRs represent dual TCRα transcripts of either MAIT or iNKT cells. Consequently, we reannotated polyclonal αβ T cells as MAIT or iNKT cells if they contained additional TRAV1-2 or TRAV10 transcripts, respectively, as summarized in Fig. 4e.

To further investigate the TCR usage, we analyzed TCR Jα and Vβ combinations among canonical TCRα-expressing MAIT and iNKT cells (Fig. 4f). We found that iNKT cells displayed a strict canonical pairing of TCR Vα10, Jα18 and Vβ25-1, reflecting a highly conserved selection process. By contrast, MAIT cells exhibited developmental differences in TCR usage, with immature MAIT cells showing less frequent use of TCR Vβ6 and Vβ20 compared with their mature counterparts. This observation suggests that canonical combinations of MAIT TCRs are selectively favored during the final stages of maturation, potentially ensuring functional optimization and long-term survival of mature MAIT cells.

Overall, these findings highlight the critical role of MR1 or CD1d recognition in defining the specificity of canonical TCRα chains. Moreover, our data suggest that MAIT cells undergo a process of clonal selection during maturation, favoring specific TCR combinations that may be crucial for their functional stability and survival.

### The diverse repertoire of MAIT and iNKT cells is shaped in the thymus

**Fig. 5:**
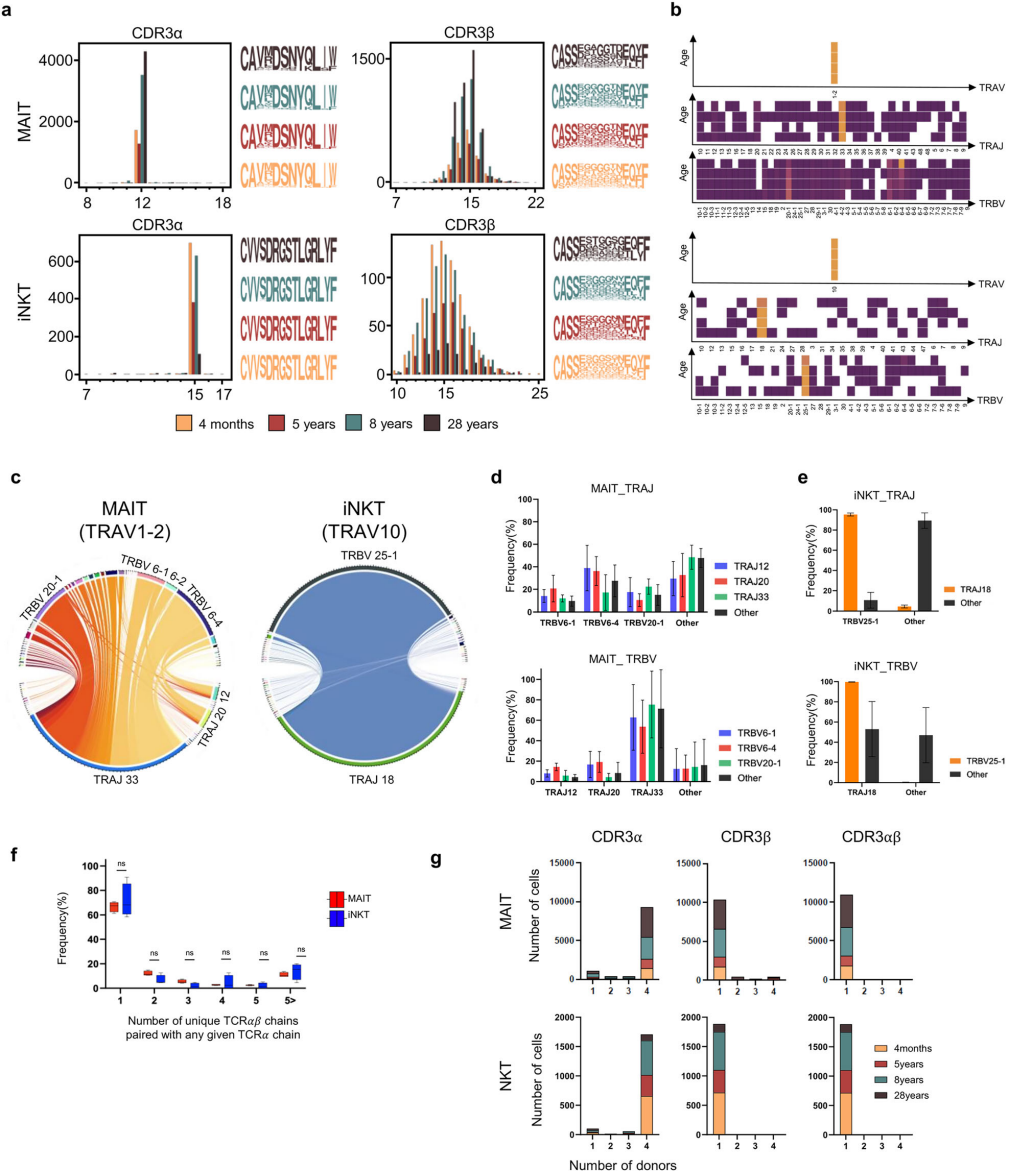
The diverse repertoire of MAIT and iNKT cells is shaped in the thymus. **a** Graphs show the distribution of CDR3α/β amino acid lengths for the indicated cell types and their corresponding amino acid sequences. **b** Heat maps illustrate TCR gene usage in each donor. **c** Chord diagrams depict the connectivity patterns between TRAJ and TRBV in MAIT and iNKT cells. **d**, **e** Graphs show the frequencies of each TRAJ and TRBV combination in MAIT (**d**) and iNKT (**e**) cells. **f** The graph shows the frequencies of cells having unique TCRαβ chains paired with any given TCRα chain. **g** Graphs show the number of shared clones of MAIT and iNKT cells between donors based on CDR3α, CDR3β or the CDR3αβ combination. Error bars indicate s.e.m.

The above findings show that the diversity of MAIT and iNKT cells arises from oligoclonal TCRβ chains paired with canonical TCRα chains. To further investigate their TCR usage, we analyzed the lengths and sequences of CDR3 loci (Fig. 5a). As expected, the canonical TRAV genes in MAIT and iNKT cells exhibited uniform CDR3α lengths with highly conserved sequences, whereas their CDR3β regions displayed significant diversity in both lengths and sequences (Fig. 5a), present in both mature and immature populations (Supplementary Fig. 12). Furthermore, no significant changes were observed in the preferential usage of TCR genes across different age groups (Fig. 5b). The chord diagram illustrates that the combinations of TCR Jα and Vβ usage are highly conserved in both MAIT and iNKT cells (Fig. 5c).

A previous study has shown that TRAJ usage influences the pairing between TRAV and TRBV chains of MAIT cells in PBMCs^5^. We also observed that TRAJ12 and TRAJ20 preferentially bind to TRBV6-4 in the thymus (Fig. 5d). In iNKT cells, which predominantly utilize TRAJ18, TRAJ chains other than TRAJ18 exhibited minimal pairing with TRBV25-1 (Fig. 5e). Moreover, the promiscuous pairing of canonical TCRα chains, previously described in the periphery^5^, was also detected in the thymus, where approximately 30% of MAIT cells exhibited more than one pairing with TCRβ chains (Fig. 5f). Although not statistically significant, iNKT cells showed a more limited pattern of pairing, consistent with their strict usage of canonical TCRs (Figs. 4e and 5f).

In both MAIT and iNKT cells, CDR3α nucleotide sequences were public, shared across all four donors (Fig. 5g, top left) in both immature and mature cells (Supplementary Fig. 13). Conversely, CDR3β sequences were private (Fig. 5g, top middle), and the combinations of CDR3α and CDR3β were not shared between donors (Fig. 5g, top right), aligning with previously described peripheral diversity in human MAIT cells^5^. These patterns were also observed in iNKT cells (Fig. 5g, bottom), confirming that the diversity of iNKT cells is similarly shaped.

Collectively, these results indicate that the diverse repertoire of MAIT cells is shaped during thymic development rather than by peripheral selection, and iNKT cells follow similar trends.

### MAIT cells clonally expand in the adult human thymus

**Fig. 6:**
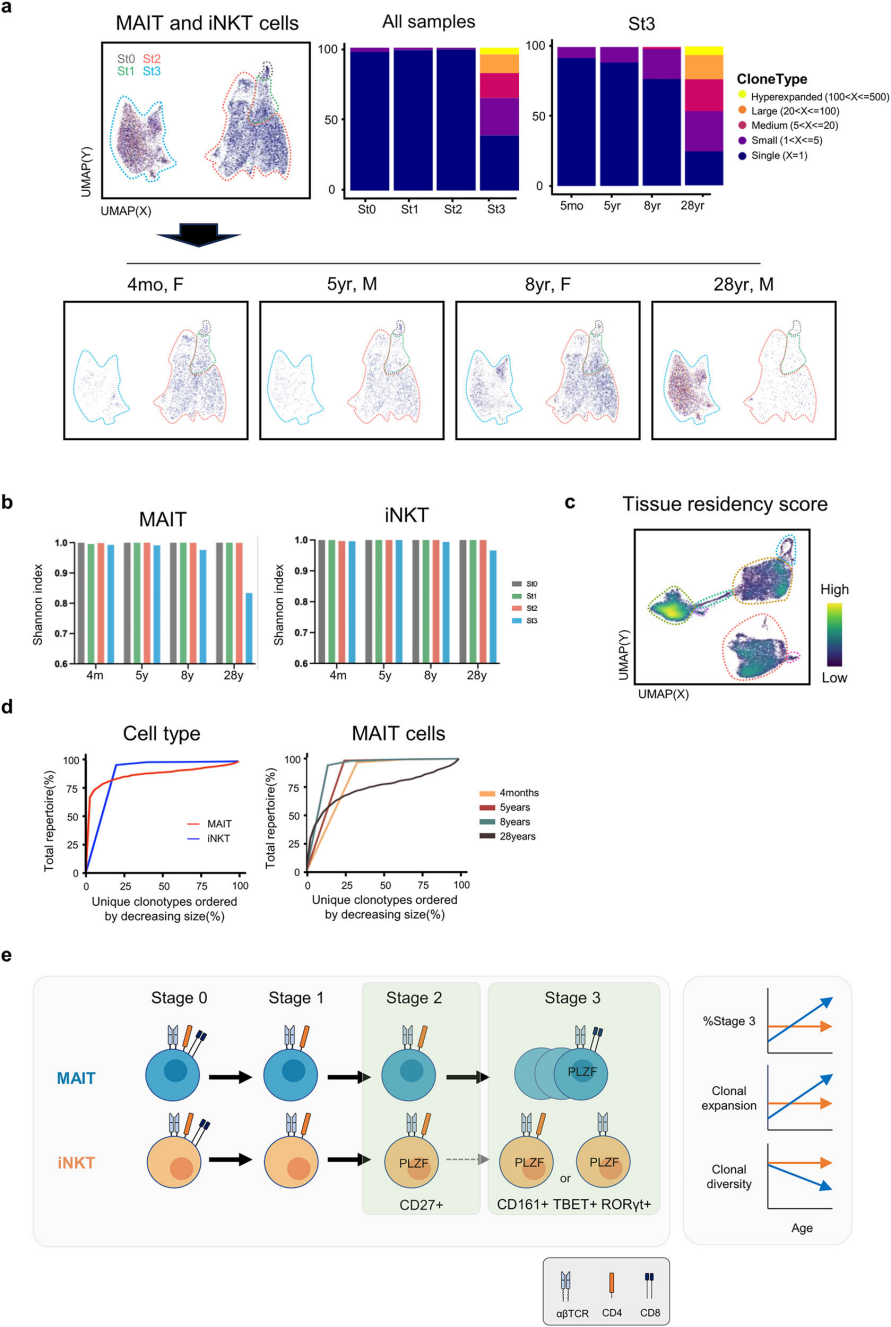
MAIT cells clonally expand in the adult human thymus. **a** UMAP plots show the distribution of the repeated clonotypes in total (top, right) and each donor (bottom) among total MAIT and iNKT cells. The bar graphs show the fraction of repeated clones in each stage (top, middle) and donor (top, right). **b** The graphs show Shannon index illustrating TCR diversity in each donor and cell type. **c** The density plot shows the tissue residency score. **d** Line plots show the clonality colored by cell type (left) and donor (right). **e** The figure summarizes the proposed model of thymic development of human unconventional T cells.

Finally, we investigated the clonal expansion of MAIT and iNKT cells in the thymus (Fig. 6). A clonotype is defined as a group of cells sharing identical nucleotide sequences across six CDR regions, indicating their derivation from a single progenitor cell. We observed that nearly all cells in stages 0–2, and approximately half of the cells in stage 3, were clonally unique, indicating that immature cells do not proliferate after positive selection (Fig. 6a). Notably, the fractions of single clonotypes showed a decreasing trend with aging (Fig. 6a, top right). Furthermore, certain clonotypes expanded over 100-fold in stage 3, predominantly originating from the thymus of a 28-year-old donor (Fig. 6a, top middle). Consequently, the Shannon diversity index, which measures clonal diversity, was significantly reduced in stage 3 MAIT cells from this donor. A slight reduction in the Shannon index was also observed in stage 3 MAIT cells from an 8-year-old donor, confirming the result in multiple donors (Fig. 6b). Importantly, these findings are consistent with our flow cytometric analysis, which showed that stage 3 MAIT cells expand in proportion to aging (Fig. 1c). Stage 3 MAIT and iNKT cells exhibited higher tissue residency scores than their immature counterparts or classical γδ T cells (Fig. 6c) across all donors (data not shown). The clonal expansion of MAIT cells was correlated with aging, whereas iNKT cells showed a clonal pattern similar to MAIT cells in young MAIT donors (Fig. 6d). In summary, our results indicate that mature MAIT cells undergo clonal expansion in the human thymus. A comprehensive summary of our findings is shown in Fig. 6e, which illustrates the distinct maturation patterns of MAIT and iNKT cells in the human thymus.

## Discussion

In this study, we conducted an extensive analysis of the developmental landscape of MAIT cells across different ages, examining their associations with iNKT cells and classical γδ T cells in the human thymus. We found that mature MAIT cells clonally expand in the human thymus, and they are distinct from iNKT cells in several features: iNKT cells express PLZF and CD4 at immature stages and progress to stage 3 less efficiently. In addition, we defined markers CD27 (not CD4) and CD161 as representing the maturation stages of iNKT cells. Our data also showed that the diverse repertoire of MAIT and iNKT cells is established in the thymus by pairing public TCRα with private oligoclonal TCRβ chains in the immature stage. We also provide evidence that MAIT cells undergo clonal selection upon maturation and proliferation with aging in the thymus, leading to the accumulation of expanded clonotypes in adult donors. Overall, these results provide new insights into the developmental landscape of human MAIT cells in the thymus.

We demonstrated that stage 3 MAIT cells underwent robust clonal expansion in the thymus of a 28-year-old donor and, to a lesser extent, in an 8-year-old donor (Fig. 6). Our findings are consistent with demographic data that used multiple samples indicating that stage 3 CD8 SP MAIT cells expand within the thymus (Fig. 1c). This suggests that the clonal expansion of mature MAIT cells may be an age-associated phenomenon, potentially reflecting their role in the adaptive immune response as individuals age. We also found that the frequency of stage 1 MAIT cells among total thymocytes remains constant in adults (Fig. 1c). These features are in contrast to the finding that peripheral MAIT cells decrease after adulthood (Fig. 1f and ref. ^43^). CD8 expression facilitates MR1 tetramer binding^44^, raising the technical issues of detecting mature MAIT cells. However, it is unlikely that this happens only in aged donors. Rather, it is possible that the homeostatic expansion of MAIT cells in the blood is less robust than that of conventional T cells or that MAIT cells become more tissue resident and do not leave the thymus in adults. Mature murine thymic NKT cells persist in the thymus^16^, and our data suggest human MAIT cells have similar trends, suggesting they form a resident memory population, which warrants further investigation into their biological significance.

While our study provides detailed insights into the thymic ontogeny of MAIT cells, several limitations should be noted. The limited number of adult thymic samples restricts our ability to fully capture age-related dynamics in single-cell analysis with statistical significance. Although stage 3 MAIT cell expansion was prominent in a donor aged 28 and, to a lesser extent, in the 8-year-old donor, further validation in elderly individuals is needed. In addition, while we considered TRAV1-2- and TRAV10-positive cells as MAIT and iNKT cells, respectively, functional validation for these TCRs binding to tetramers remains necessary to confirm their repertoire selection during maturation (Fig. 4e, f).

MAIT and iNKT cells share developmental markers yet exhibit divergent maturation and clonal expansion patterns, particularly with aging. Only MAIT cells undergo robust clonal expansion and acquire tissue-residency signatures in adults. One speculation on these differences is that human thymi continuously provide ligands for MAIT TCRs, while such ligands for iNKT cells are limited. In contrast to humans, murine NKT1 cells in the thymus exhibit similar features, becoming long-lived tissue-resident populations^16^, whereas MAIT cells are rare in mice. The differences in self or nonself ligands expressed in the thymus could be a leading factor determining cell-type-specific residency in different species.

Functionally, MAIT cells contribute to antimicrobial defense and are implicated in chronic inflammation and autoimmune diseases^45^. Their capacity to produce IFN-γ and IL-17 and persist as tissue-resident populations positions them as key players in mucosal immunity. One potential implication of clonally expanded mature MAIT cells in the adult thymus is that they might provide inflammatory cytokines to developing immature conventional T cells in the thymus, potentially conditioning them to become more responsive in the periphery. This speculation requires further investigation in the future.

Previous studies on MAIT cells either did not annotate TCR information^8,9^ or excluded all T cells having dual TCRα transcripts^5^. So far, the nature of dual TCRα transcripts or polyclonal repertoire of MAIT and iNKT cells has not been thoroughly explored. Here, we demonstrated that most polyclonal TCRs do not bind to MR1 or CD1d tetramers (Fig. 4c and Supplementary Fig. 11). This suggests that the expression of canonical TCR transcripts of MAIT and iNKT cells is sometimes surpassed by those of polyclonal TCRs. In theory, dual TCRα transcripts are present in about 30–35% of human T cells, resulting from the simultaneous recombination of two TCRα alleles following β-selection, which occurs sequentially with stringent allelic exclusion^46–48^. Given that the detection rate of TCRα sequences in scRNA-seq is approximately 70%, the frequency of dual TCRα transcripts detected in single-cell sequencing is calculated to be ~19–22%. A recent meta-analysis found that 15% of human thymocytes express dual TCRα transcripts^41^, and our result was 16.2% (corresponding to 25% in the actual value), falling between the theoretical and observed values of conventional T cells. This suggests that human MAIT and iNKT cells are derived from DP thymocytes undergoing random TCR rearrangement, similar to murine iNKT cells^49^. Quality control results also support that our findings are not due to the incomplete exclusion of doublets, and we estimate the contamination frequency to be less than 5% when assessing the proportions of polyclonal cells lacking canonical TCR expression (Supplementary Fig. 4).

A previous study has shown that iNKT cells are rare in postnatal human thymus and almost undetectable using anti-TCR antibodies^50^. However, recent studies using CD1d tetramer-based enrichment have provided insight into the transcriptional characteristics of iNKT cells in both the human thymus^8,9,51^ and blood^52^. These studies revealed transcriptional similarities between iNKT and MAIT cells, and our research further identified unique features of iNKT and γδ T cells, highlighting the heterogeneity of human unconventional T cells. In mice, the absence of iNKT or γδ T cells leads to a threefold increase in MAIT cell numbers^12,53^, suggesting a mutually suppressive relationship. In humans, it remains unclear whether the developmental arrest of iNKT and γδ T cells at stage 2 in the thymus is influenced by MAIT cells or if these cells require extrathymic environments for final maturation.

Collectively, our findings suggest that human MAIT cells undergo clonal selection and expansion during thymic maturation unlike other types of innate T cell. These characteristics align more closely with conventional T cells selected by agonistic self-peptides despite the unique ability of MAIT cells to recognize 5-OP-RU, a metabolite derived from intestinal bacteria. These insights provide a deeper understanding of the mechanisms underlying thymic selection and the shaping of the T cell repertoire of unconventional T cells.

## Supplementary information

Supplementary Information

Supplementary Table 1

Supplementary Table 2

## Data Availability

Sequencing data generated in this study have been deposited in NCBI’s Gene Expression Omnibus (GEO) and are accessible through GEO SuperSeries accession number GSE254975. The MSigDB database and WebLogo generator are publicly available.

## Code availability

No custom software was generated for this manuscript. The code developed for the analyses performed in this study is available upon request. The software used for all analyses is listed in the relevant sections of the ‘Materials and methods’.
