# Supplementary material for: Human MAIT cells undergo clonal selection and expansion during thymic maturation and aging
Source: Exp Mol Med. 2025 Aug 8;57(8):1789–801. doi: 10.1038/s12276-025-01509-x (PMC12411618; doi:10.1038/s12276-025-01509-x)
Supplement: Supplementary file 1 — Supplementary Information [file 12276_2025_1509_MOESM1_ESM.pdf]

Supplementary Information

Human MAIT cells undergo clonal selection and expansion during thymic maturation and aging

Myeong-seok Lee, Su Yeong Park, Jung-Hwan Choi, Seon Yong Bae, Ryu Han Suk, Min-Sung Kim, Jae Gun Kwak and You Jeong Lee

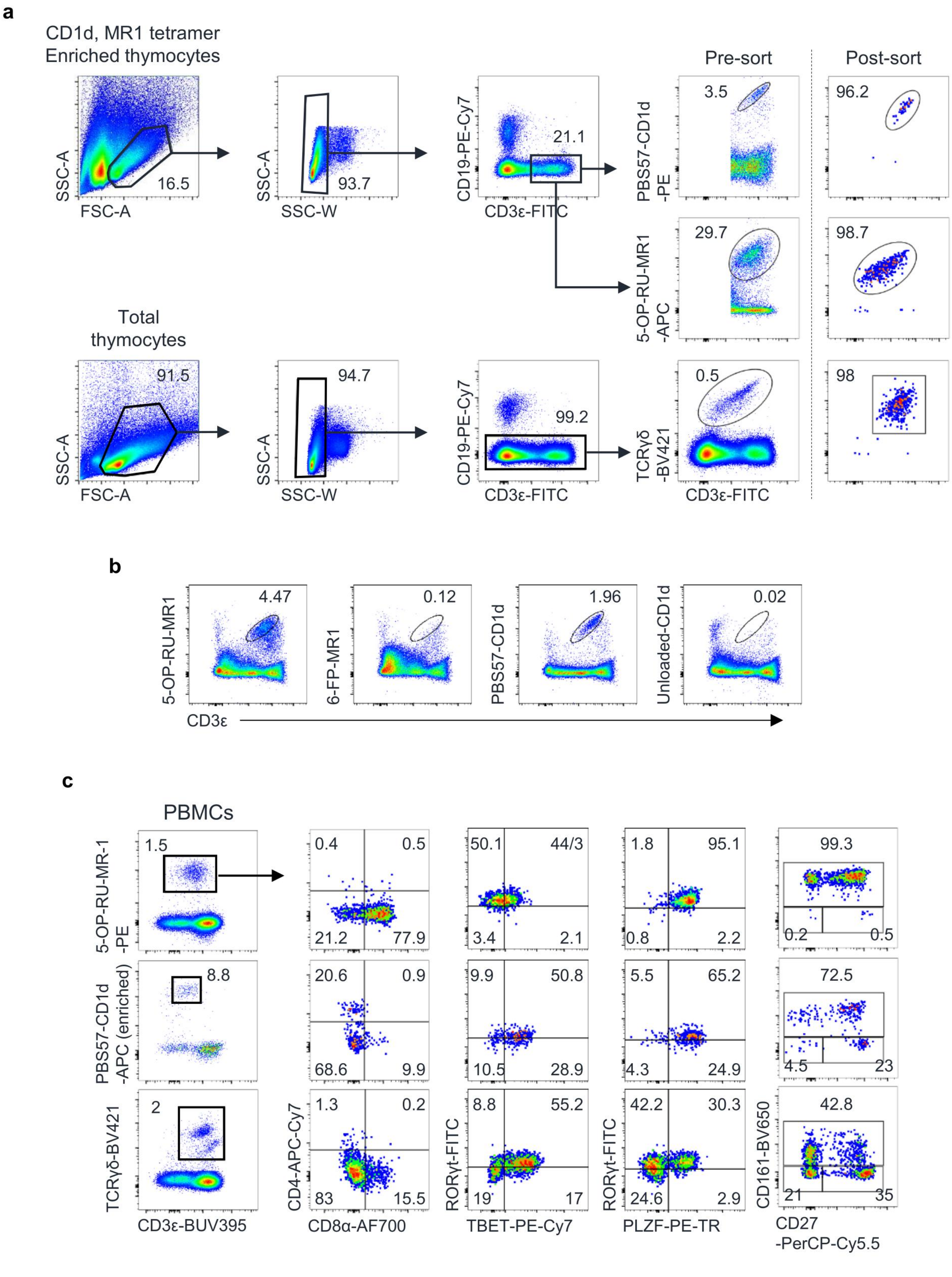

Supplementary Figure 1. Analysis of human unconventional T cells in the thymus. (a) Dot plots depict the sorting strategy of iNKT, MAIT and γδT cells (left, pre-sort) and the sorting purity of each cell type used for single-cell RNA sequencing (right, post-sort). (b) Representative facs plots show total thymocytes enriched with 5-RO-RU-loaded MR1, 6-FP-loaded MR-1, and PBS57-loaded or unloaded CD1d tetramers. (c) Each unconventional T cell was analyzed from human PBMCs. iNKT cells were enriched using PBS57-loaded CD1d tetramers, while MAIT and γδ T cells were detected from total cells. Dot plots show representative facs data from at least 3 independent experiments. Numbers indicate frequencies of cells in adjacent gates or quadrants.

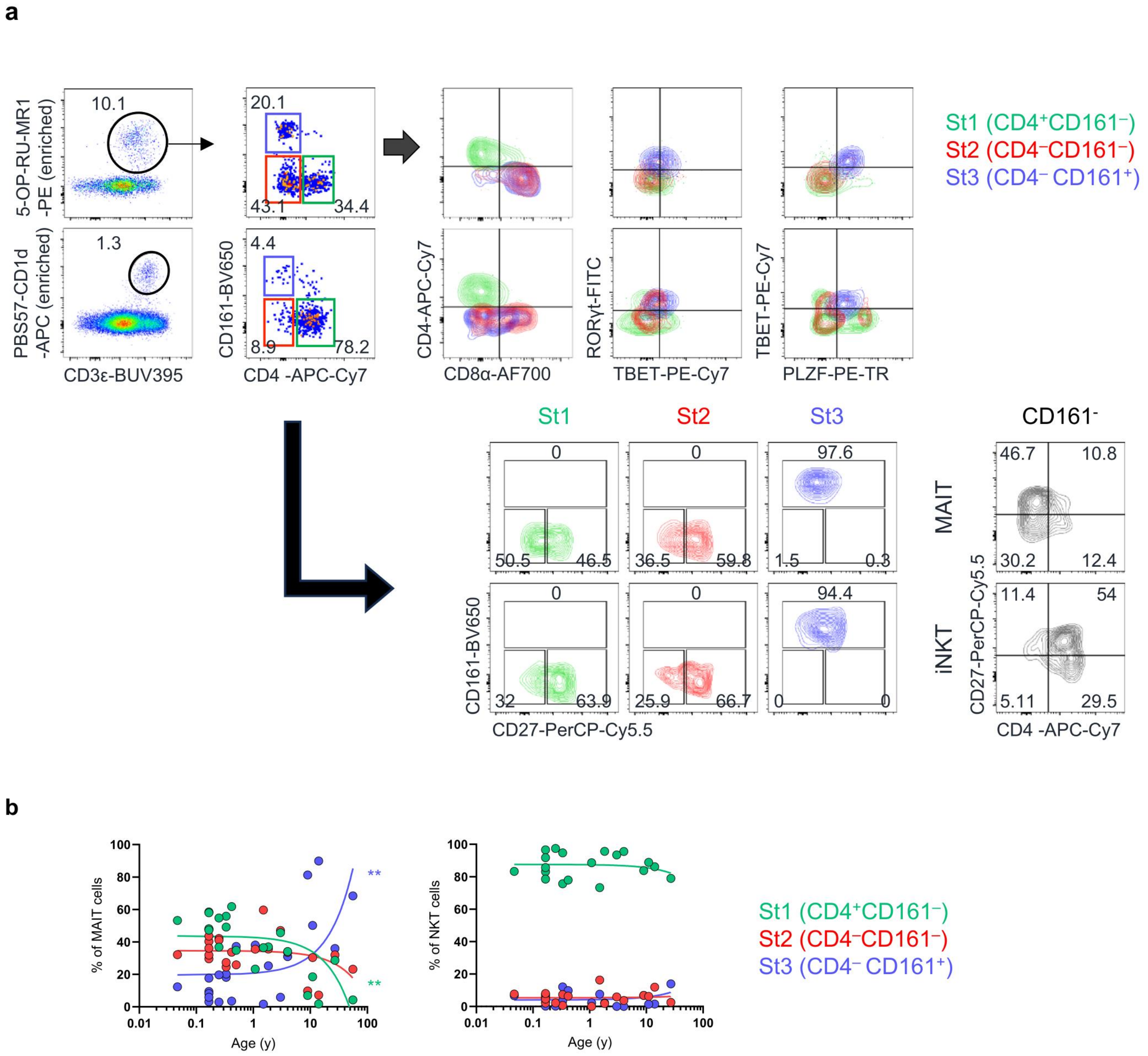

Supplementary Figure 2. Facs analysis of human thymic MAIT and iNKT cells. (a) Represent dot and contour plots show the phenotype of each stage of MAIT (top) and iNKT (bottom) cells using CD4 and CD161 and are compared with CD27 and CD161. (b) Graphs show the age-dependent kinetics of each stage of MAIT (left) and iNKT (right) cells. **P* < 0.05, ***P* < 0.01, ****P* < 0.001.

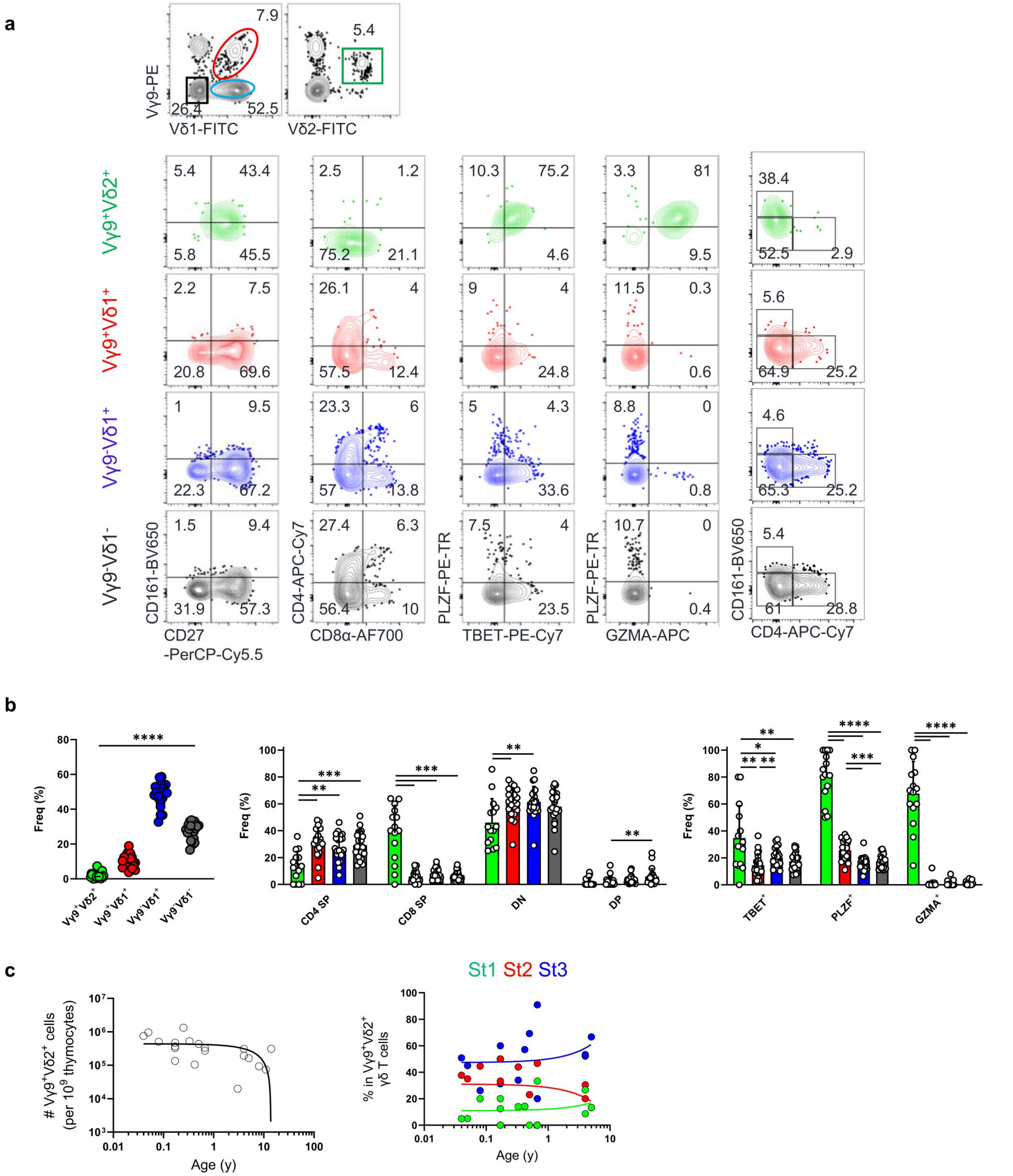

Supplementary Figure 3. Vγ9⁺Vδ2⁺ γδ T cells represent innate T cells in the thymus. (a) Representative dot plots show the expression of indicated markers in four different γδ T cell subsets as depicted in different colors. (b) Graphs illustrate the phenotype of each γδ T subset. An ANOVA analysis was used. (c) Graphs show the total number of Vγ9⁺Vδ2⁺ γδ T cells (left) and frequencies of each stage (right). *P < 0.05, **P < 0.01, ***P < 0.001.

a

Quality Control
Step 1. Remove unqualified cells based on individualized criteria for pDoublet, nFeature_RNA, percent.mito.
Step 2. Exclude non-target cells based on cell marker expression, such as B cells, DCs, etc.
Step 3. Qualify gdT cells with TRDC expression > 0.5 and no TCRαβ chain sequence expression.
Step 4. Validate αβ TCR cells to ascertain the presence of the TRAC and TRBC genes

b

Age	Sex	pDoublet	nFeature_RNA(x)	percent.mito
4months	Female	<0.7	200< x < 6000	<10
5months	Male	<0.7	100 < x < 6000	<6
8years	Female	<0.7	100 < x < 6000	<6
28years	Male	<0.7	100< x < 5000	<10

Cell markers				
T	CD3E	CD4	CD8A	PTPRC
MAIT	TRAV1-2	TRBV20-1		
NKT	TRAV10	TRBV25-1		
gdT	TRDC	TRDV1	TRGC2	
B	CD19	MS4A1	PAX5	
DC	ITGAX			

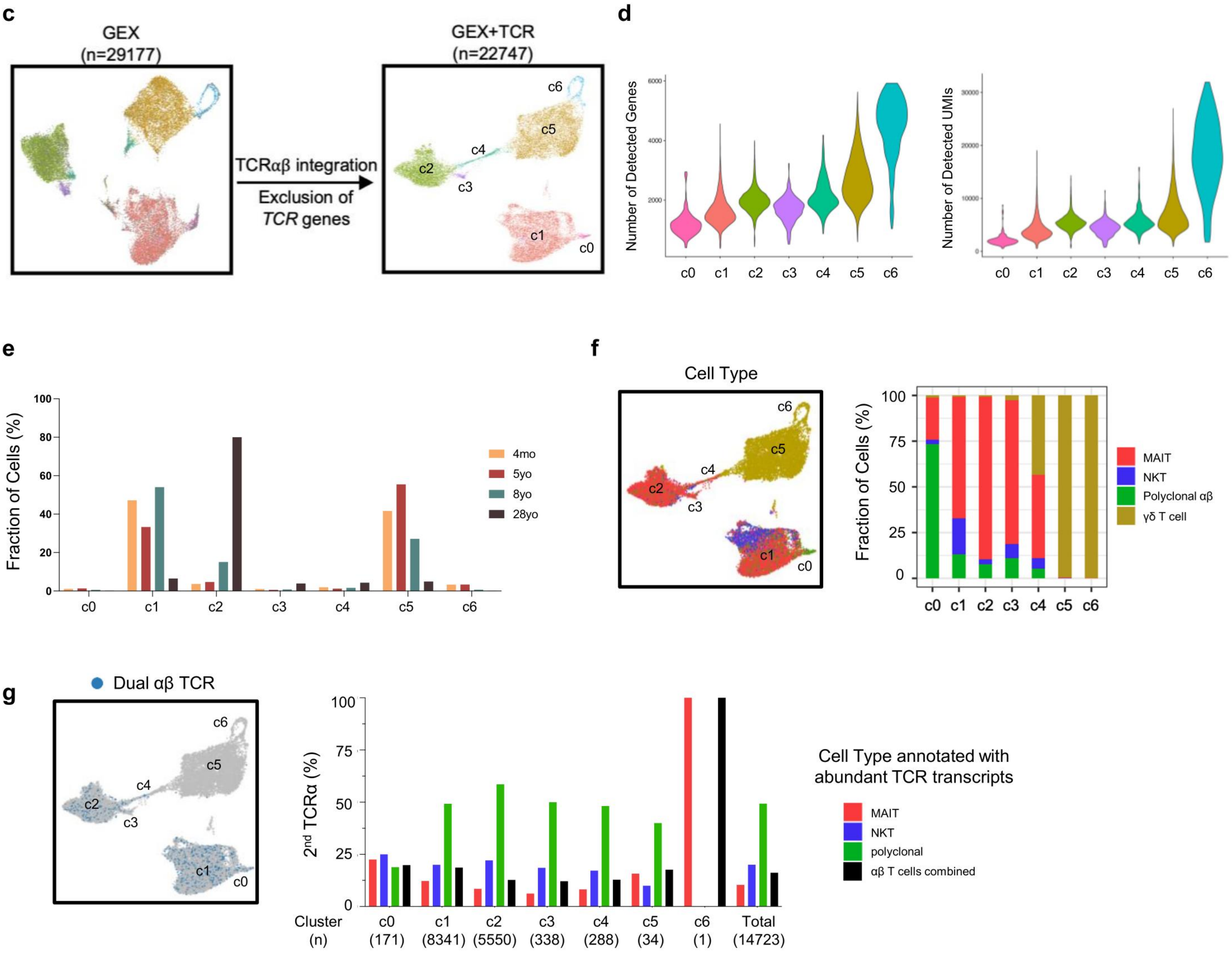

Supplementary Figure 4. Quality control for scRNA-seq. (a) Workflow summary of quality control performed on each sample for single cell analysis. (b) Tables summarize the parameters used to filter low-quality cells for each sample (top), and the markers employed to exclude non-target cells (bottom). (c) UMAP representation of plots before (left) and after (right) scTCR-seq data integration. (d) Violin plots illustrate the number of detected genes (left), and UMIs (right) per cell for each cluster. (e) Bar graph displays the fraction of cells per sample for each cell cluster. (f) A UMAP plot shows the distribution of cell types (left). Bar graph displays the fraction of cell types designated per each cluster (right). With dual TCRα transcripts, cell types were annotated based on the transcript with higher expression. (g) UMAP plot shows the distribution of cells with dual TCRα transcript (left). Bar graph displays the percentage of cells that have 2nd TCRα in each cluster for each cell type (right).

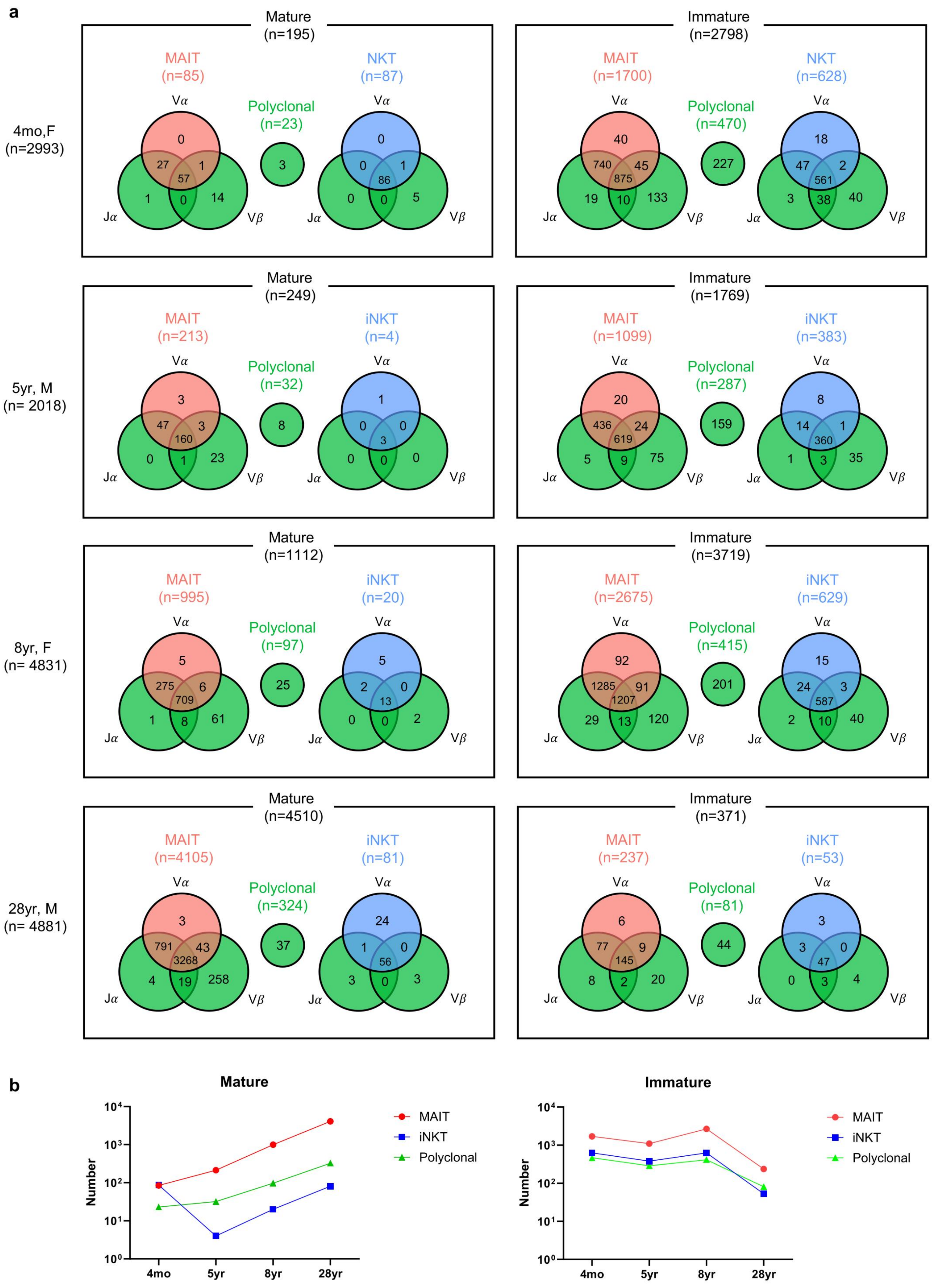

Supplementary Figure 5. Clonotyping analysis of αβ T cells. Venn diagrams (a) and graphs (b) show the number of cells with indicated TCR combinations in each sample.

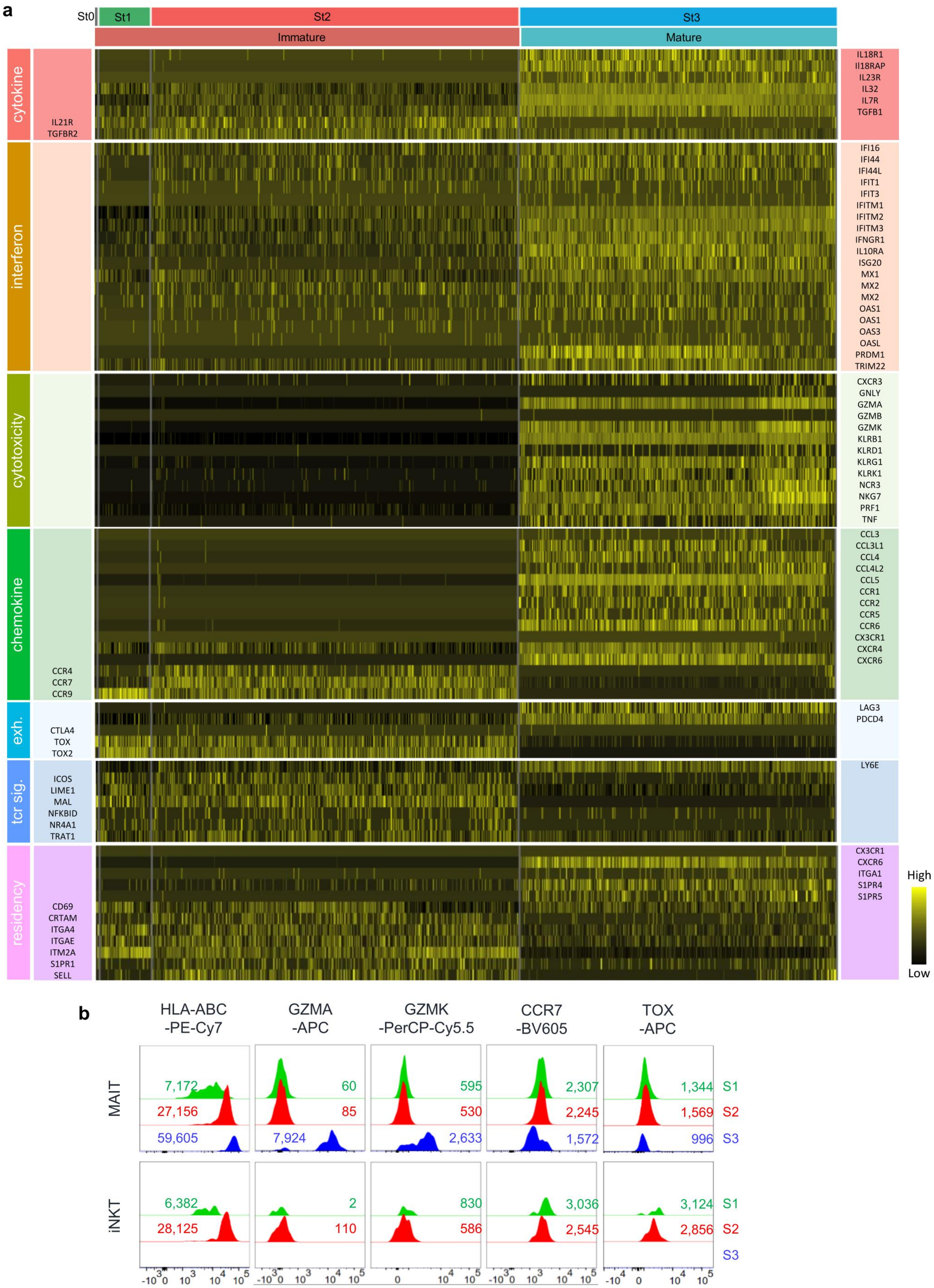

Supplementary Figure 6. Stage-specific signature gene expression pattern. (a) A heatmap shows DEGs associated with maturation states based on functionality-driven criteria. (b) Histograms show the FACS analysis of the indicated markers across developmental stages of MAIT (top) and iNKT (bottom) cells.

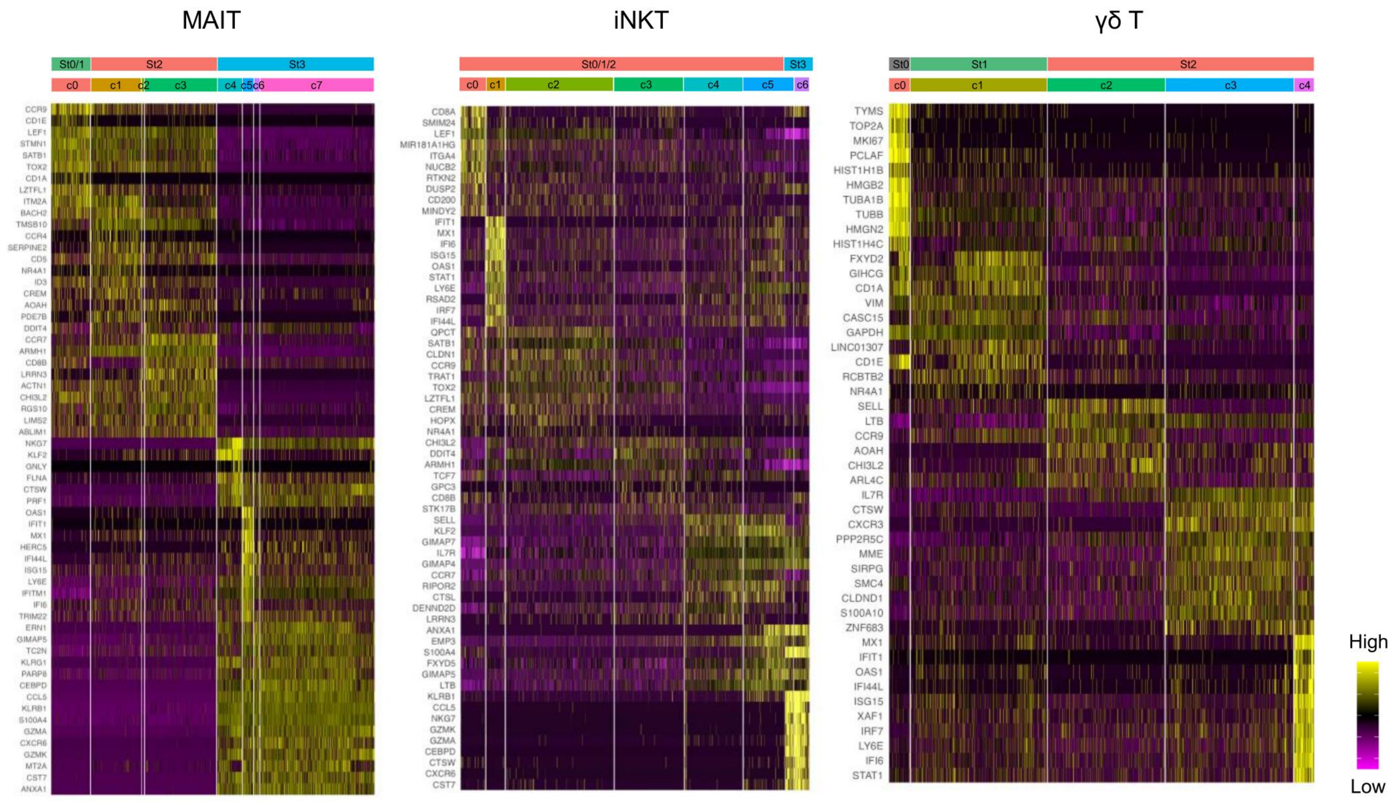

Supplementary Figure 7. Heatmaps show differentially expressed genes in each cell type.

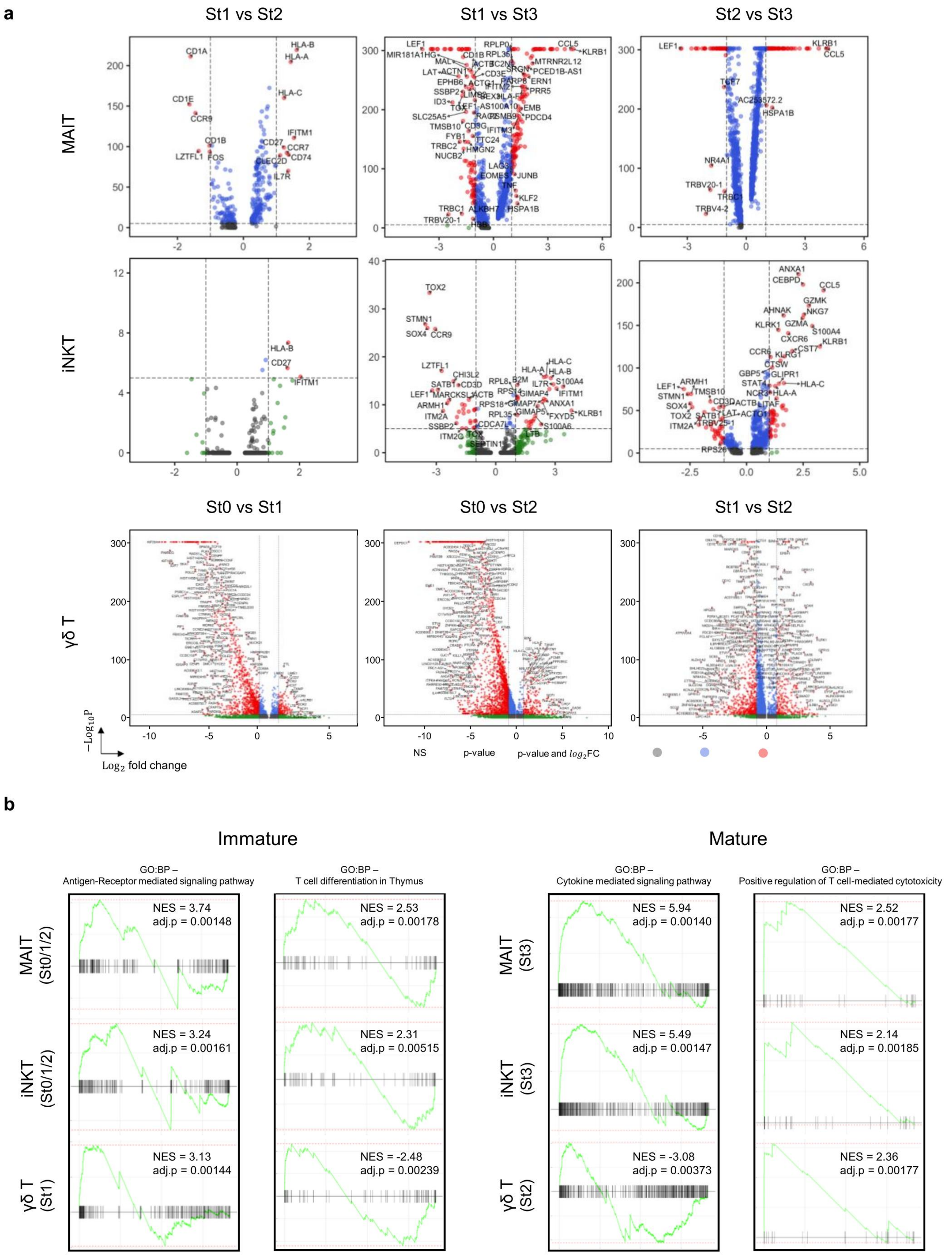

Supplementary Figure 8. Comparison between stages of MAIT, iNKT and γδ T cells. (a) Volcano plots show the differentially expressed genes (DEGs) between maturation stages in indicated populations. Pathway analyses reveal shared and unique pathways of each cell type. (b) GSEA plots show enriched pathways in immature and mature populations.

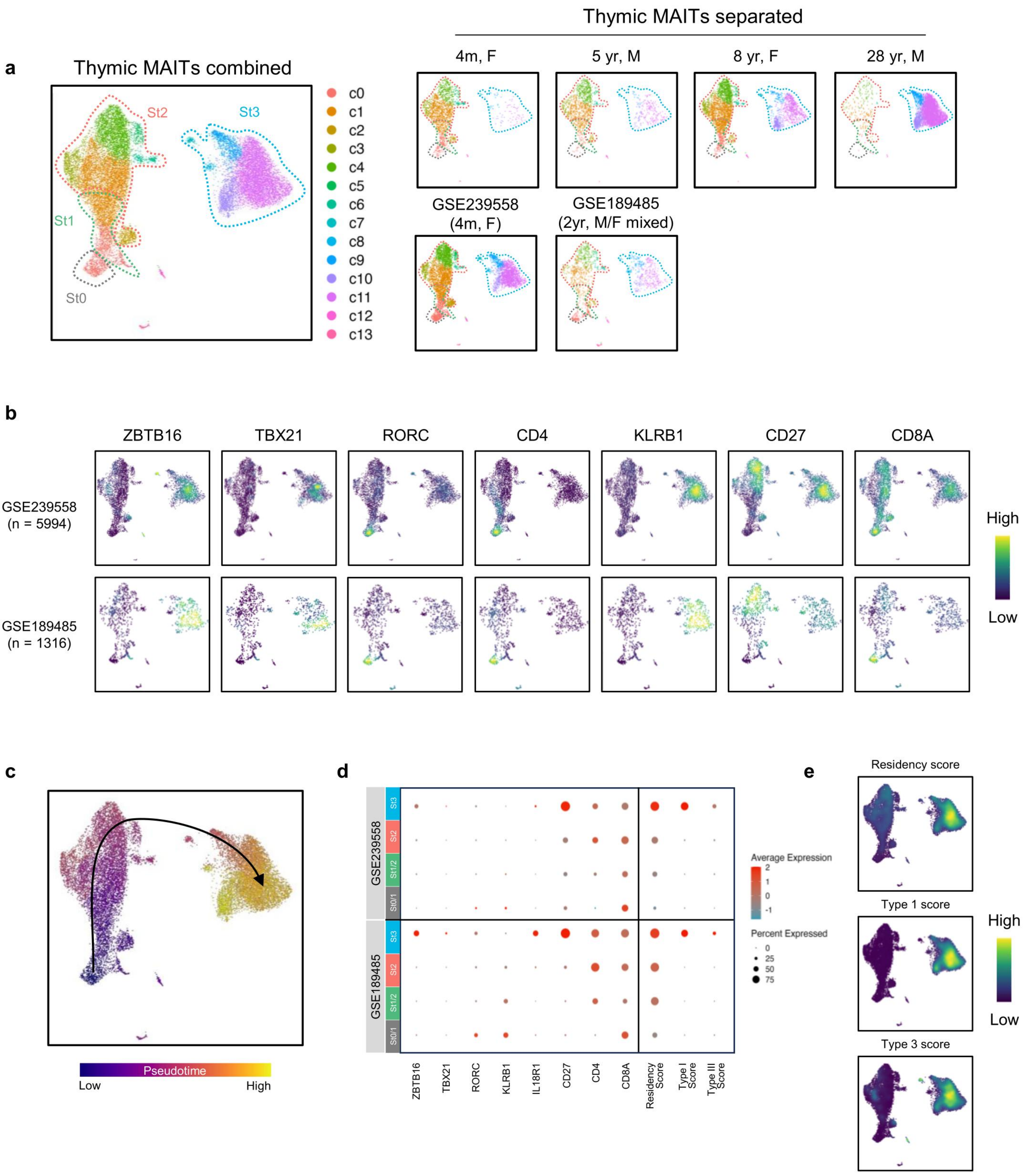

Supplementary Figure 9. Combined analysis of MAIT cells with public data sets. (a) A UMAP plot shows MAIT cells combined with two public data sets (left) and UMAP plots in each data set (right). (b) Feature gene plots show the expression of indicated markers in two public data sets. (c) Pseudotime analysis of combined UMAP plot. (d) Dot plot shows the expression of indicated markers in two public data sets. (e) Density plots show residency and type 1/3 scores in combined UMAP.

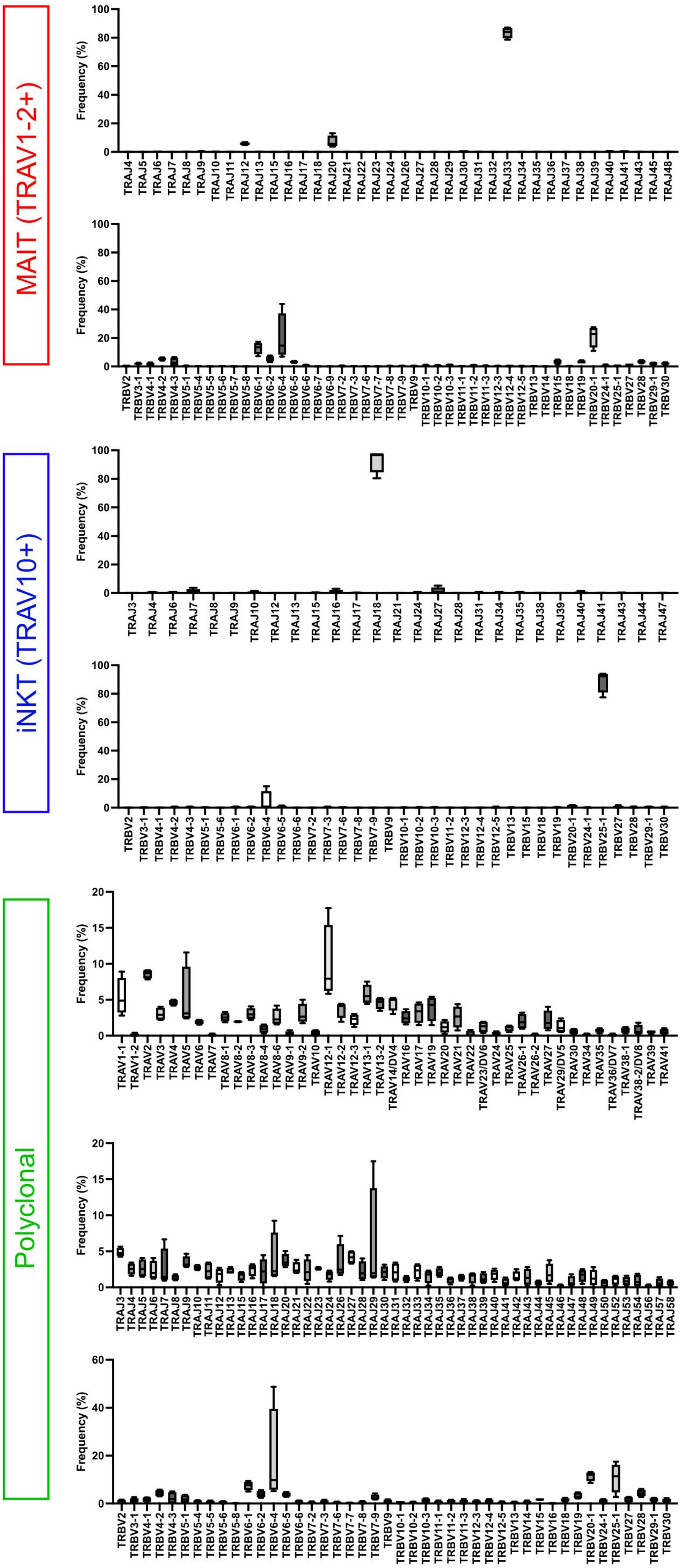

Supplementary Figure 10. TCR gene usage pattern of αβ innate T cells. Graphs show the frequencies of indicated TCR gene usage in MAIT, iNKT, and polyclonal cells. Error bars indicate SEM.

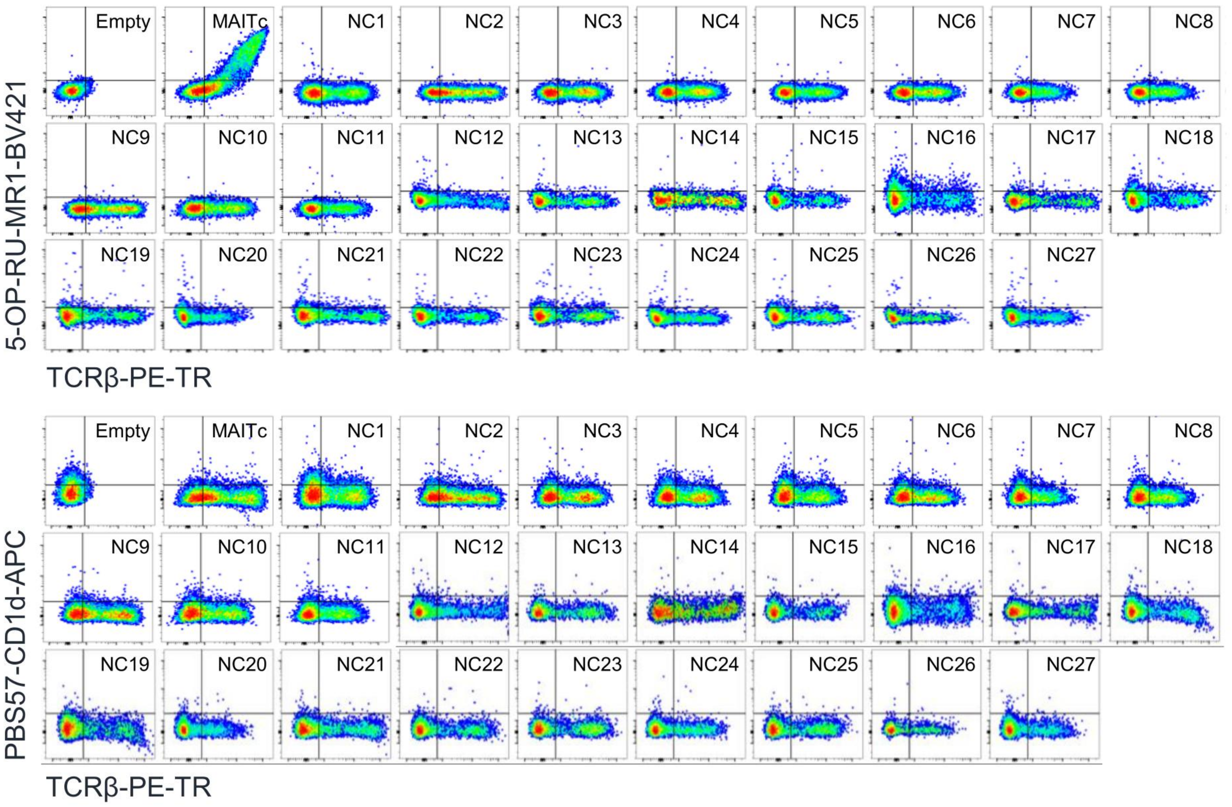

Supplementary Figure 11. Polyclonal TCRs recognize neither MR1 nor CD1d. HEK293T cells were transfected with 27 polyclonal TCRs (NC #1~27), canonical MAIT TCR (MAITc), or empty vectors and stained with MR-1 and CD1d tetramers.

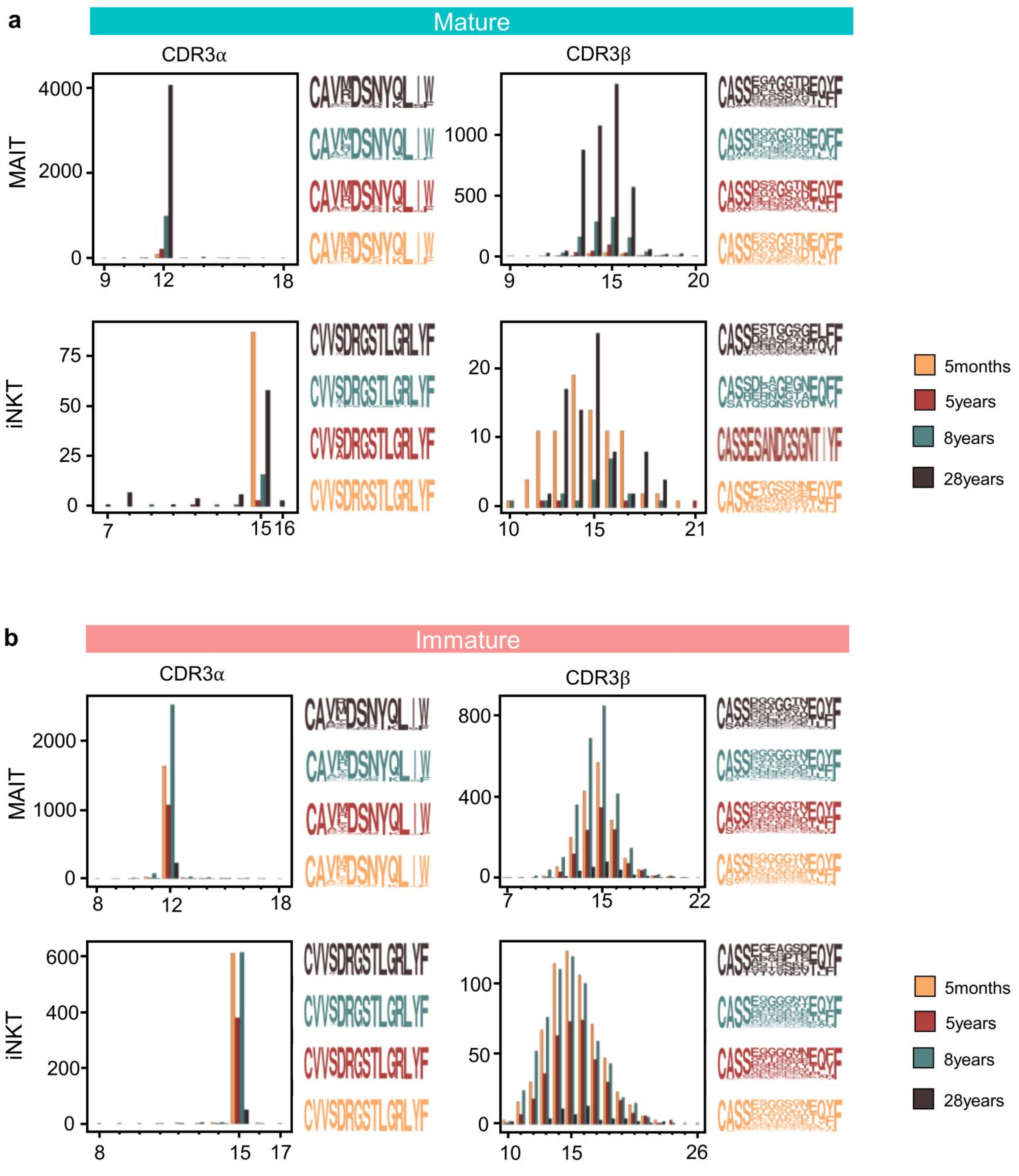

Supplementary Figure 12. CDR3 diversity of αβ innate T cells. Graphs show the amino-acid length distribution of CDR3α and CDR3β regions in mature (a) and immature stages (b) with variation of amino acid sequences within the highest peak.

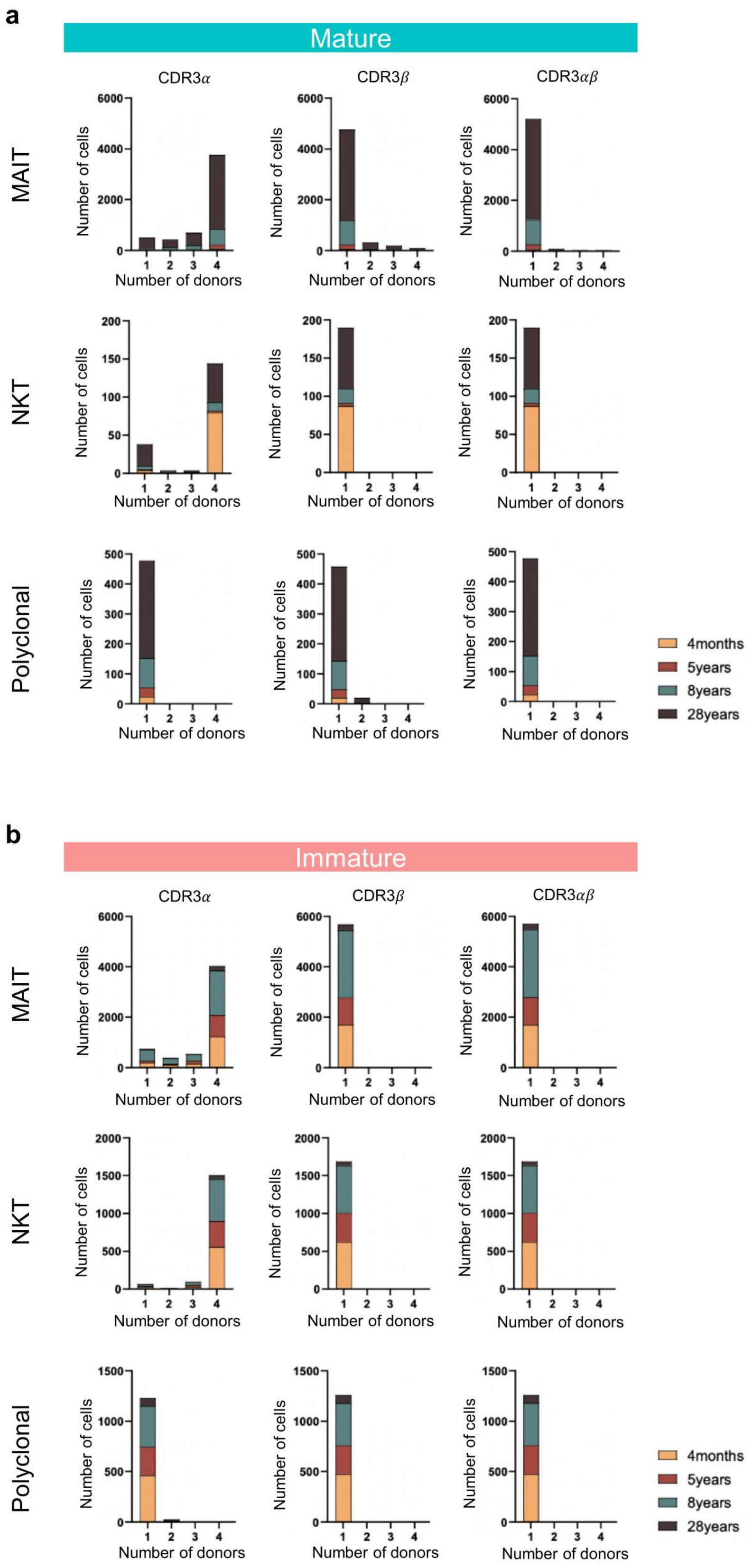

Supplementary Figure 13. Canonical CDR3α sequences are shared between samples. Graphs show the number of shared clones between samples in mature (a) and immature (b) populations based on CDR3α, CDRβ, or CDR3αβ.
